# CDC42 expression is altered by dioxin exposure and mediated by multilevel regulations via AhR in human neuroblastoma cells

**DOI:** 10.1038/s41598-017-10311-3

**Published:** 2017-08-31

**Authors:** Tuan Xu, Heidi Q. Xie, Yunping Li, Yingjie Xia, Yangsheng Chen, Li Xu, Lingyun Wang, Bin Zhao

**Affiliations:** 1 State Key Laboratory of Environmental Chemistry and Ecotoxicology, Research Center for Eco-Environmental Sciences, Chinese Academy of Sciences, Beijing, 100085 China; 2 University of Chinese Academy of Sciences, Beijing, 100085 China

## Abstract

Emerging evidence has shown that dioxin causes dysregulation of microRNAs (miRs) in a variety of tissues or cells. However, little is known about dioxin effects on neuronal miRs expression. In the present study, 277 differentially expressed miRs were identified by miRs microarray analysis in 2,3,7,8-tetrachlorodibenzo-p-dioxin (TCDD, at 10^−10^ M) treated SK-N-SH neuroblastoma cells. Among them, 53 miRs exhibited changes of more than 0.4-fold. Consistent with the microarray data, we verified the induction effect of TCDD on hsa-miR-608 expression, which is a primate-specific miR associated with brain functions. Bioinformatics analysis showed involvement of hsa-miR-608 in cytoskeleton organization, in which one of the hsa-miR-608 target genes, Cell Division Cycle 42 (CDC42), might play a role. We also confirmed induction of CDC42 expression by TCDD in SK-N-SH cells. TCDD induced the expression of CDC42 mRNA in hsa-miR-608 inhibitor transfected cells more obviously than in control cells, suggesting involvement of both transcriptional and post-transcriptional mechanisms in the TCDD-induced CDC42 regulation. Furthermore, CH223191, an antagonist of the aryl hydrocarbon receptor (AhR), counteracted TCDD-induced hsa-miR-608 and CDC42 expression. These results indicated that AhR not only mediates transcriptional induction of CDC42, but also hsa-miR-608-induced post-transcriptional regulation of CDC42 in dioxin treated neuroblastoma cells.

## Introduction

Exposure to dioxins or dioxin-like compounds (DLCs) has been associated with impaired development of the nervous system and loss of brain functions (e.g. motor coordination, emotion, cognition and psychological disorders) in experimental animals and humans^1–5^. Dioxins cause alterations in expression of various genes, including neurotransmission^6, 7^, neurodevelopment^8, 9^ and cytotoxicity related genes^10, 11^, which are considered as important mechanisms for dioxin effects. The aryl hydrocarbon receptor (AhR) is known as a key mediator of transcriptional regulation of the altered gene expression caused by dioxin exposure^6, 8^, in which dioxin binds to the AhR to form the dioxin-receptor complex, undergoes nuclear translocation and coupling with AhR nuclear translocator, and finally interacts with the dioxin responsive elements (DREs) in promoters of target genes to manipulate gene expression^12^. With the recent rise of epigenetics, emerging evidence suggested possible involvement of dioxins or DLCs in epigenetic regulation^13^. However, whether this epigenetic regulation by dioxins could be present and participate in the adverse effects on the nervous system remains unclear.

As one of the major epigenetic mechanisms, microRNAs (miRs), a new class of small non-coding RNAs, post-transcriptionally regulate gene expression by inhibiting mRNA translation or inducing mRNA degradation^14^. The miRs participate in diverse aspects of neuronal development, function and plasticity^14, 15^. Their dysfunction may lead to neurodevelopmental abnormalities and neurodegenerative disorders^15^. Brain-enriched miR-124 increases neuron formation through the repression of SRY-box transcription factor Sox9 during neurogenesis in adult mice, and promotes neurite elongation through the inhibition of ROCK1 expression in M17 cells^16, 17^. A primate specific miR, miR-608, inhibits the migration and invasion of glioma stem cells by targeting macrophage migration inhibitory factor^18^, and altering its expression in the brain could affect anxiety^19^.

Dioxin has been demonstrated to cause dysregulation of miRs in a variety of tissues or cells, such as miR-191-5p in HepG2 cells^20^, miR-335-5p in MDA-MB-231 and BT474 cells^21^, miR-212-3p and miR-132-3p in MDA-MB-231 and BT474 cells^22^, miR-25-3p and miR-92a-3p in multiple myeloma cells^23^. However, little is known about the effect of dioxins on miR expression in the human nervous system. Given the important function of miRs in the nervous system and the above correlation between dioxin and miRs, we hypothesize that gene alterations caused by dioxins in the nervous system may be due to both transcriptional and post-transcriptional mechanisms involving miRs.

A human neuroblastoma cell line, SK-N-SH cell, exhibits numerous biochemical characteristics of neurons, and has been employed as a model of human neurons to investigate the toxicity of dioxins^6, 24^. In the current study, we adopted miR microarray analyses to perform global analysis of differentially expressed miRs caused by dioxins. To make the study relevant to real environmental pollution, an average concentration of dioxin in human serum was used as the experimental treatment concentration^6^. Aberrant expression profiling of miRs was obtained in dioxin-treated SK-N-SH cells. Among them, a primate-specific miR was selected for further validation and function study. A target gene of this identified miR was chosen to reveal the effect of dioxin on its expression and underling mechanism for the dysregulation of the gene, including the role of AhR in regulation.

## Results

### Alteration in miRs expression profile in response to TCDD in SK-N-SH cells

**Figure 1:**
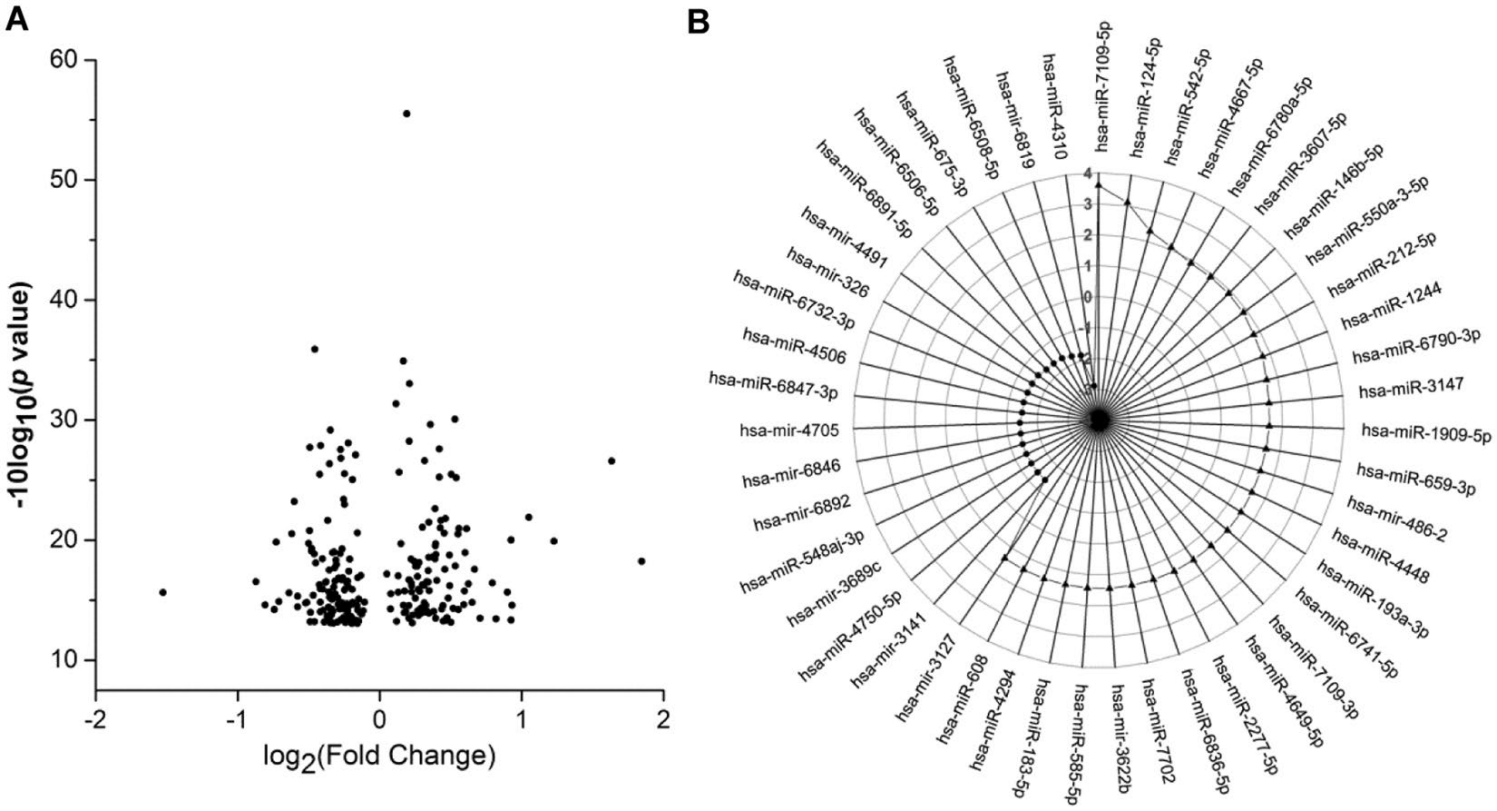
Differentially expressed miRs in response to dioxin treatment in SK-N-SH cells. Cells were treated with 10^−10^ M TCDD or 0.1% DMSO for 24 hr. (**A**) The volcano plot shows the −10log_10_ (p-value) on the y-axis and the fold of change (log_2_) on the x-axis. (**B**) A radar chart presenting 53 miRs exhibiting more than 0.4-fold of changes, including 32 up-regulated miRs and 21 down-regulated miRs.

For an overview of the effects of dioxin on the expression of neuronal miRs, we analyzed the miR expression profiles using Affymetrix GeneChip miR 4.0 Arrays in SK-N-SH cells treated with 2,3,7,8-tetrachlorodibenzo-p-dioxin (TCDD) (at 10^−10^ M) or DMSO. The clustered heat map showed discrimination among all the treatment groups (Supplementary Fig. 1). In addition, a dendrogram of the cluster analysis of miRs was generated based on the similarity between different treatment groups (Supplementary Fig. 1). The biological replicates were clustered tightly together and separated clearly from the other treatment suggesting that the differences between the two groups were mainly due to the different treatments (Supplementary Fig. 1). A total of 277 miRs were differentially expressed in SK-N-SH cells treated with 10^−10^ M TCDD as compared with DMSO-treated cells with a cut-off of p < 0.05 (Fig. 1A and Supplementary Table 1). Among these differentially expressed miRs, 53 miRs exhibited more than 0.4-fold of changes, including 32 up-regulated miRs and 21 down-regulated miRs (Fig. 1B). According to previously published studies, 13 of these 53 miRs are related to the nervous system, including the primate specific hsa-miR-608 and human specific hsa-miR-183-5p (Table. 1). To our knowledge, dioxin-induced alterations in the expression of hsa-miR-608 or hsa-miR-183-5p have not been documented in human-derived neuronal systems.

**Table 1:** Association of 13 differentially expressed miRs with pathological conditions in the nervous system.

Disease/cell lines	miRBase: Accession	miR	Reference
Brain tumors	MIMAT0004762	hsa-miR-486-3p	31
	MIMAT0004591	hsa-miR-124-5p	32
	MIMAT0000756	hsa-miR-326	33–36
	MIMAT0000459	hsa-miR-193a-3p	37
	MIMAT0007882	hsa-miR-1909-5p	38
	MIMAT0003276	hsa-miR-608&	18
	MIMAT0002809	hsa-miR-146b-5p	39–41
	MIMAT0000261	hsa-miR-183-5p#	42, 43
Neurodegenerative diseases	MIMAT0002177	hsa-miR-486-5p	44
	MIMAT0002809	hsa-miR-146b-5p	45–48
	MIMAT0000459	hsa-miR-193a-3p	47, 48
	MIMAT0018006	hsa-miR-3622b-3p	49
	MIMAT0019711	hsa-miR-4649-5p	50
Tumors cell lines	MIMAT0004591	hsa-miR-124-5p	32
	MIMAT0002809	hsa-miR-146b-5p	39, 40, 51
	MIMAT0000756	hsa-miR-326	36, 52–54
	MIMAT0003276	hsa-miR-608	18, 55
	MIMAT0003340	hsa-miR-542-5p	56
	MIMAT0003337	hsa-miR-659-3p	57
	MIMAT0000261	hsa-miR-183-5p	42

Note: & represents primate specific miR, ^#^ represents human specific miR.

### Dioxin induces the expression of hsa-miR-608

**Figure 2:**
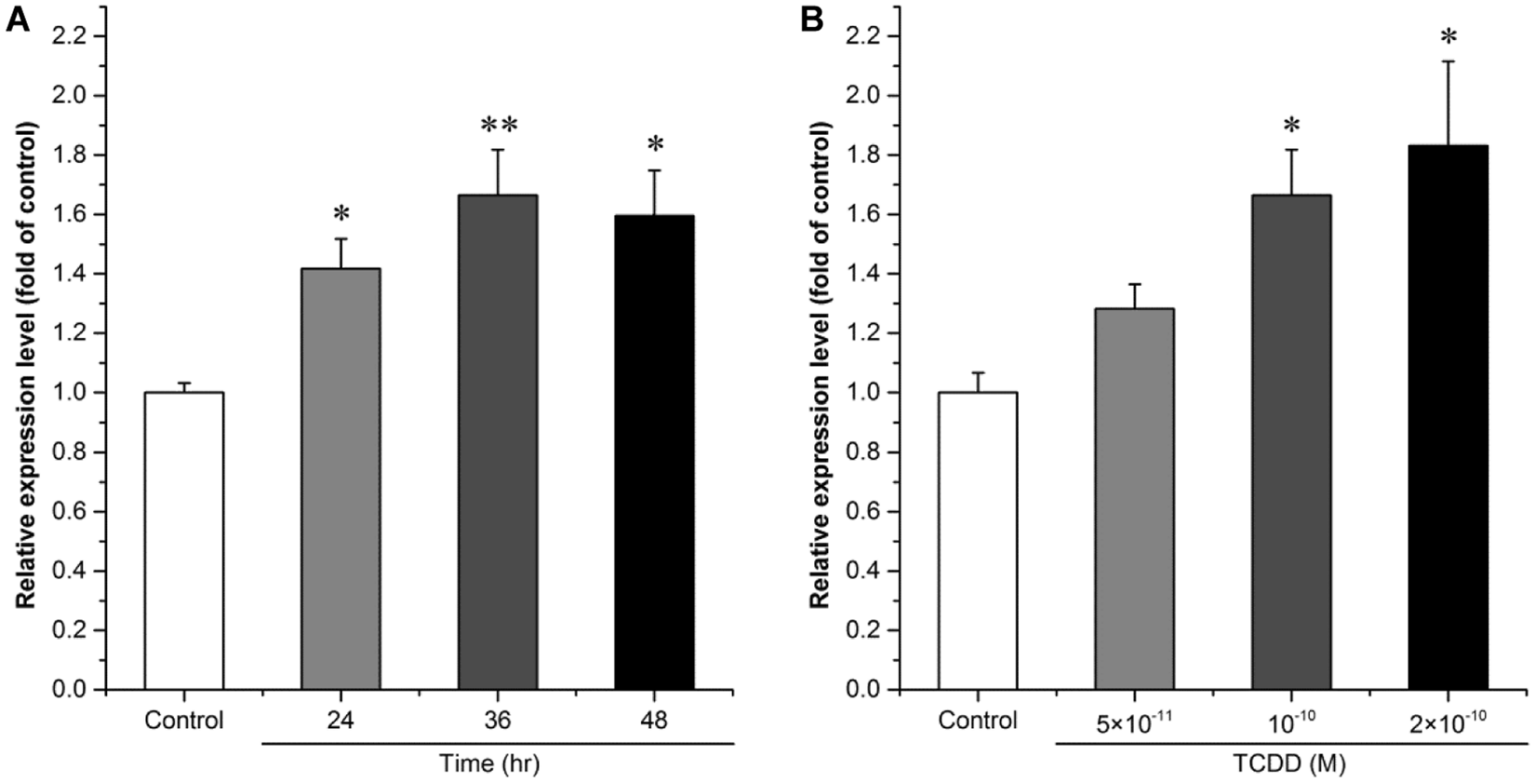
Effect of TCDD on the expression of hsa-miR-608 in cultured SK-N-SH cells. SK-N-SH cells were treated with 10^−10^ M TCDD or 0.1% DMSO for 24 hr, 36 hr or 48 hr (**A**), or with 5 × 10^−11^, 10^−10^, or 2 × 10^−10^ M TCDD or 0.1% DMSO for 36 hr (**B**). Total miRs was extracted for determination of the expression level of hsa-miR-608. Quantitative PCR analyses were performed as mentioned in M & M. U6 rRNA was used as an internal control. Values were expressed as mean ± S.E. (n = 3) and each independent sample was detected in triplicate. Statistical analysis was done by t-test (**A**) or by one-way ANOVA with Bonferroni test (**B**), and *p < 0.05, **p < 0.01, compared with control (DMSO treated cells).

The effect of TCDD on hsa-miR-608 expression was further validated by quantitative PCR (qPCR) analysis^25^. We found the expression of hsa-miR-608 was significantly increased by approximately 42%, 67% and 50% after treatment with 10^−10^ M TCDD for 24, 36, 48 hr, respectively (Fig. 2A, p = 1.82E-02, p = 8.93E-06 and p = 2.85E-02, respectively). However, there were no significant differences between the effects at the different time points. Because 36 hr was the time point showing the greatest change in hsa-miR-608 expression, 36 hr was used to reveal the response of hsa-miR-608 expression to different concentrations of TCDD. Results showed that the expression of hsa-miR-608 was significantly increased by approximately 83% after 36 hr treatment with 2 × 10^−10^ M TCDD compared with DMSO treated controls (Fig. 2B, p = 1.59E-02). However, the lowest testing concentration of TCDD (5 × 10^−11^ M) showed no effect on hsa-miR-608 expression (Fig. 2B, p = 1.000) and there was no change in the expression of the human-specific miR, hsa-miR-183-5p, upon TCDD treatment (data not shown).

### Predicted biological function of hsa-miR-608

**Figure 3:**
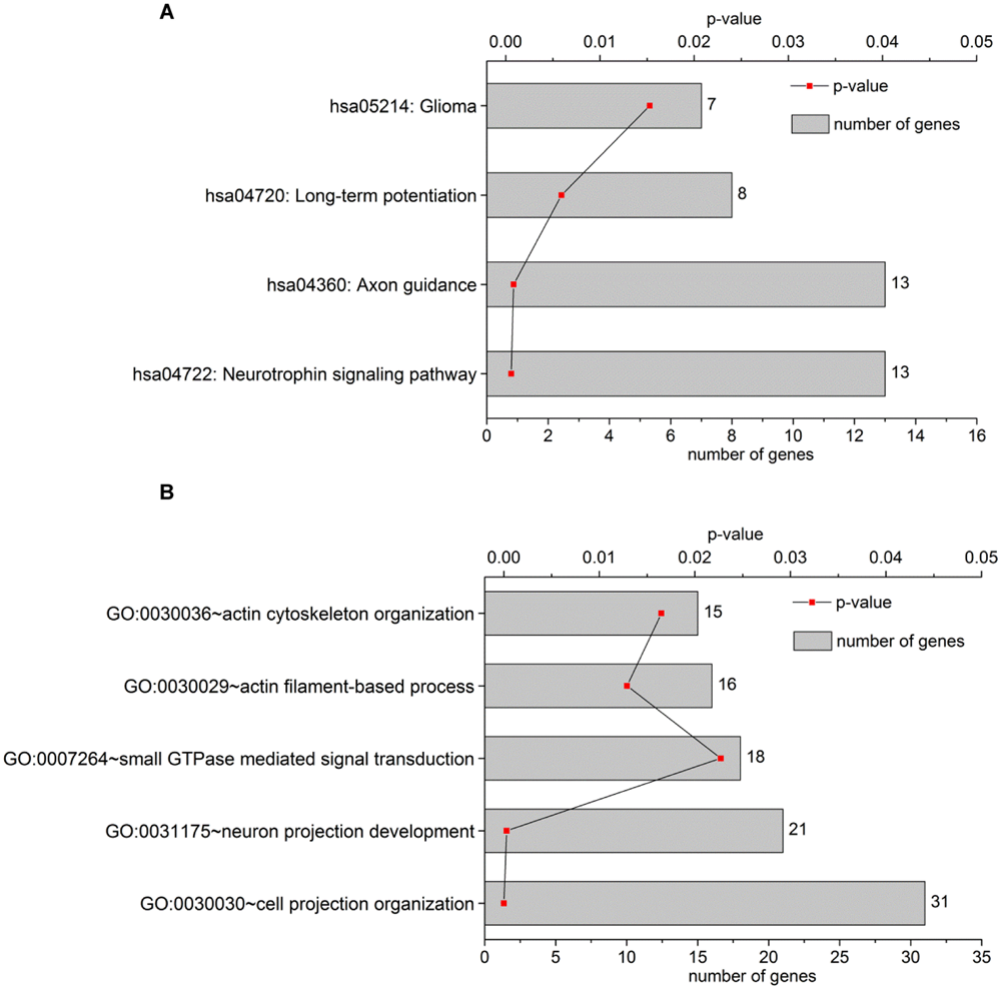
Functional annotations related to the nervous system for hsa-miR-608 target genes. KEGG pathway (**A**) and GO (**B**) cluster analyses were performed via DAVID website software. The vertical axis shows the annotated functions of the target genes. The horizontal axis shows p-value and the gene number of each cluster.

Target gene searching is important for functional predictions of miRs^14^. From the literature, 17 genes have been reported as target genes of hsa-miR-608 (Supplementary Table 2). Because of the limitation of experimentally verified target genes of hsa-miR-608, computational prediction was included in the study^26^. There were 557 communal target genes predicted for hsa-miR-608 employing two widely used online software programs (Supplementary Table 3). After removing duplicate genes, 571 unique target genes were identified and subjected to functional clustering. In total, 25 Kyoto Encyclopedia of Genes and Genomes (KEGG) pathways and 220 Gene ontology (GO) terms (including 164 Biological Processes (BP), 25 Molecular Functions (MF) and 31 Cellular Components (CC)) were found to be related to these genes using DAVID online software (Supplementary Tables 4 and 5). Based on KEGG analyses, the target genes of hsa-miR-608 were associated with several well-known neuronal signaling pathways, including hsa05214: glioma (p = 1.53E-02), hsa04720: long-term potentiation (p = 5.92E-03), hsa04360: axon guidance (p = 8.38E-04) and hsa04722: neurotrophin signaling pathway (p = 5.87E-04) (Fig. 3A). Gene Ontology analysis showed that hsa-miR-608 target genes are involved in a series of important cellular and molecular events such as GO: 0030036 ~actin cytoskeleton organization (p = 1.68E-02), GO: 0030029 ~actin filament-based process (p = 1.32E-02), GO: 0007264 ~small GTPase mediated signal transduction (p = 2.32E-02), GO: 0031175 ~neuron projection development (p = 2.96E-04) and GO: 0030030 ~cell projection organization (p = 4.16E-06) (Fig. 3B). Among the genes associated with these neural biological functions, Cell Division Cycle 42 (CDC42) was the one present with the highest frequency and selected for further study.

### Involvement of hsa-miR-608 in gene regulation of CDC42 by dioxin

**Figure 4:**
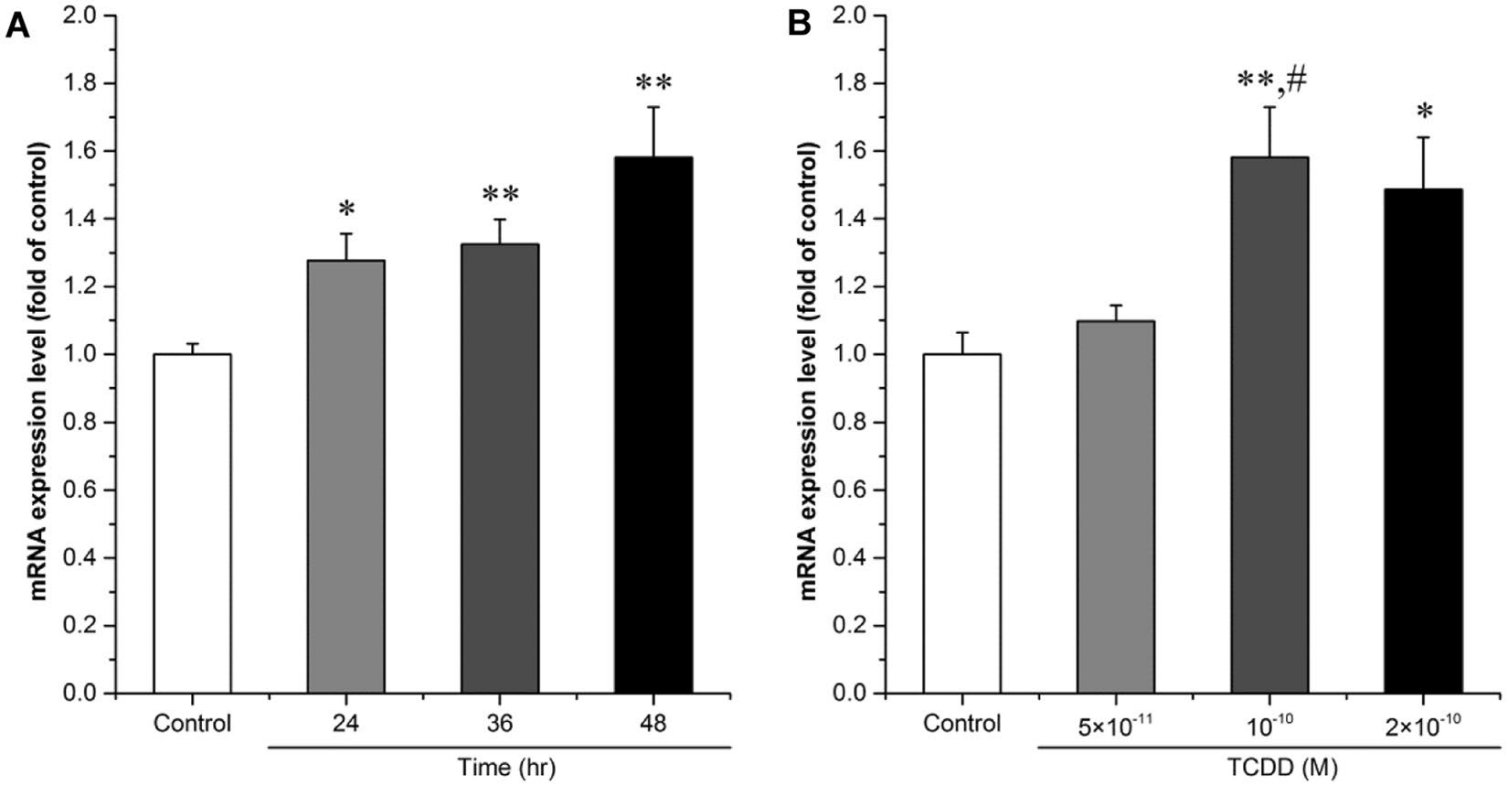
Effect of TCDD on the expression of CDC42 in cultured SK-N-SH cells. Cells were treated with 10^−10^ M TCDD or 0.1% DMSO for 24 hr, 36 hr or 48 hr (**A**), or with 5 × 10^−11^, 10^−10^, or 2 × 10^−10^ M TCDD or 0.1% DMSO for 48 hr (**B**). Expression level of the CDC42 mRNAs was determined by qPCR analysis. 18 S rRNA was used as an internal control. Values are expressed as mean ± S.E. from triplicate samples in three independent experiments. Statistical analysis was done by one-way ANOVA with Bonferroni test. *p < 0.05 and **p < 0.01, compared with control (DMSO treated cells). ^#^p < 0.01 compared with 5 × 10^−11^ M TCDD.

The effects of TCDD on the expression of CDC42 were verified in SK-N-SH cells. The expression of CDC42 gradually increased with time increase and showed significant induction by approximately 28%, 32% and 58% upon TCDD (10^−10^ M) treatment at different time points compared with the DMSO controls, respectively (Fig. 4A, p = 2.39E-02, p = 6.43E-03 and p = 2.44E-03, respectively). Because 48 hr was the time point showing the greatest change in CDC42 expression, this treatment time was used for the following experiments. The expression of CDC42 was significantly increased by approximately 49% after treatment with 2 × 10^−10^ M TCDD compared with DMSO treated controls (Fig. 4B, p = 3.58E-02). Significant change (approximately 44%) was found in the 10^−10^ M TCDD treated group compared with the DMSO treated control (Fig. 4B, p = 3.72E-02), however, the lowest concentration of TCDD (5 × 10^−11^ M) showed no effect on CDC42 expression (Fig. 4B, p = 1.000).

**Figure 5:**
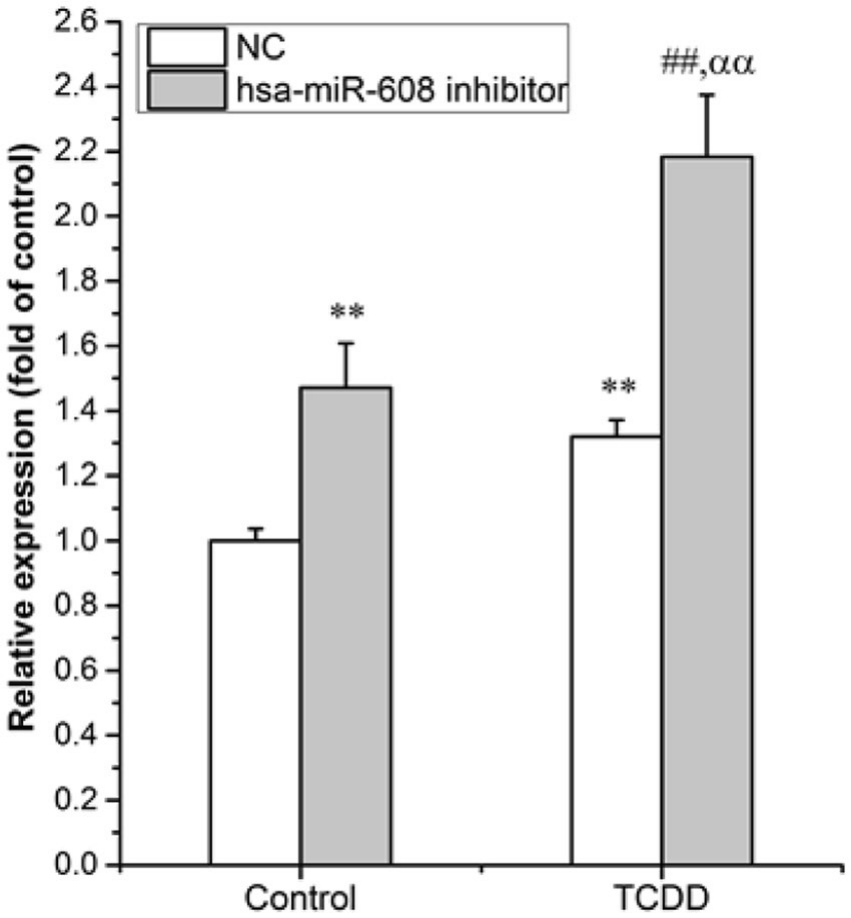
Involvement of hsa-miR-608 in gene regulation of CDC42 by dioxin. Cells were transfected with hsa-miR-608 inhibitors or NC for 24 hr, followed by 10^−10^ M TCDD treatment for an additional 36 hr. Total mRNAs was extracted for determination of the expression level of CDC42. Quantitative PCR analyses were performed as mentioned in M & M. 18 S rRNA was used as an internal control. Values are expressed as mean ± S.E. from triplicate samples in three independent experiments. Statistical analysis was done by one-way ANOVA with Bonferroni test. **p < 0.01 compared with Control (DMSO treated cells) & NC (NC transfected cells) group, ^##^p < 0.01 compared with Control (DMSO treated cells) & hsa-miR-608 inhibitor (Inhibitor transfected cells) group, ^αα^p < 0.01 compared with TCDD (TCDD treated cells) & NC (NC transfected cells) group.

In line with the finding in human breast cancer cells, the mRNA expression of CDC42 was suppressed in SK-N-SH neuroblastoma cells transfected with an hsa-miR-608 mimic (Supplementary Fig. 2, p = 3.36E-02). Moreover, the mRNA expression of CDC42 was increased in hsa-miR-608 inhibitor transfected cells compared to a negative control (NC), further supporting the notion that hsa-miR-608 can suppress gene expression of neuronal CDC42 (Fig. 5, p = 1.26E-03). To investigate the role of hsa-miR-608 in neuronal CDC42 gene regulation by dioxin, SK-N-SH cells transfected with the hsa-miR-608 inhibitor or NC were treated with TCDD or solvent. We found TCDD induced the mRNA expression of CDC42 in hsa-miR-608 inhibitor transfected cells more obviously than in NC transfected cells, suggesting involvement of hsa-miR-608 mediated suppression in the dysregulation of CDC42 by dioxin (Fig. 5, approximately 118% versus 30% of induction, p = 9.12E-10). On the other hand, in hsa-miR-608 inhibitor transfected cells, the TCDD treatment group had a significant induction effect on CDC42 mRNA expression compared to the solvent treated group, suggesting that a mechanism other than hsa-miR-608 induced suppression is involved in CDC42 regulation upon TCDD treatment (Fig. 5, approximately 118% versus 47% of induction, p = 3.23E-05). This mechanism may involve transcriptional induction of CDC42 by TCDD.

### Involvement of AhR in regulation of CDC42 by dioxin

**Figure 6:**
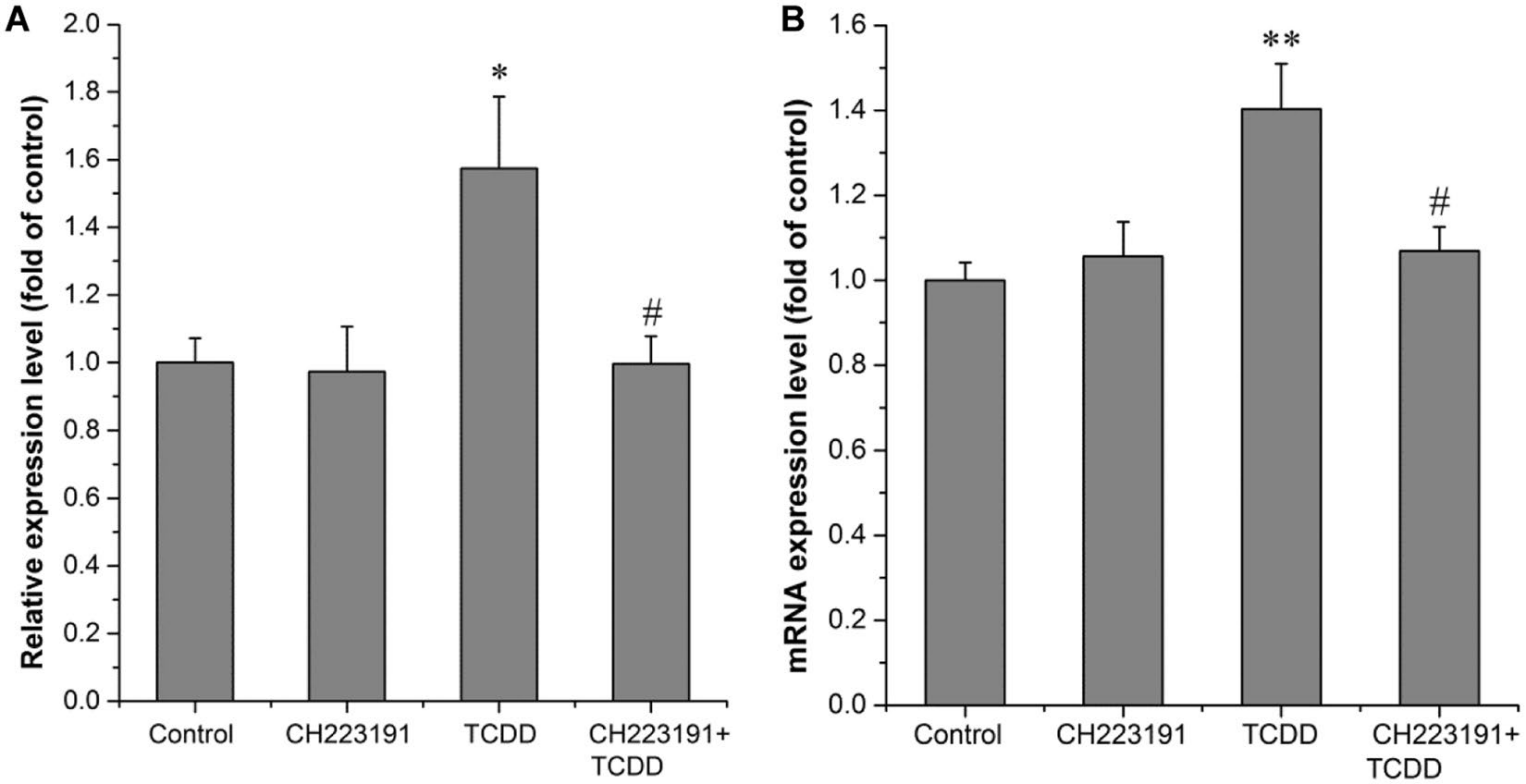
TCDD induces hsa-miR-608 and CDC42 expression via AhR-dependent pathway. Cells were treated with 10^−6^ M CH223191 (AhR antagonist) or 0.01% DMSO for 3 hr before treatment with 10^−10^ M TCDD or 0.01% DMSO for 36 hr (**A**) and 24 hr (**B**). Total miRs/mRNA was extracted for determination of the expression level of hsa-miR-608 (**A**) or CDC42 (**B**). Quantitative PCR analyses were performed as mentioned in M & M. U6 rRNA was used as an internal control for hsa-miR-608 and 18 S rRNA was used as an internal control for CDC42. Values were expressed as mean ± S.E. (n = 3) and each independent sample was detected in triplicate. Statistical analysis was done by one-way ANOVA with Bonferroni test. *p < 0.05 and **p < 0.01compared with control (DMSO treated cells). ^#^p < 0.05 compared with TCDD treatment alone.

We further explored the role of AhR in the regulation of CDC42 by dioxin. Cells were treated with CH223191, a ligand-selective antagonist of the AhR, before TCDD or solvent treatment. We found that the induction of hsa-miR-608 by TCDD was totally reversed by CH223191 treatment (Fig. 6A). Moreover, the pretreatment with CH223191 could eliminate the effect of TCDD on CDC42 expression as well (Fig. 6B). These results suggested that AhR might be a communal upstream molecule for both the transcriptional induction of CDC42 and the post-transcriptional suppression involving hsa-miR-608.

## Discussion

Dioxin is not only involved in the regulation of gene expression at the transcriptional level, but also at the post-transcriptional level, such as through miRs^20–23^. The expression of miRs can be influenced by various external environment conditions including dioxin exposure^27^. MiR microarray technology is a powerful high throughput tool capable of revealing alterations in miR expression profiles, which benefits global understanding of the post-transcriptional molecular bases of dioxin effects or toxicities^28, 29^. However, to our knowledge, there is no systematic study on dioxin induced miR alterations in models of the human nervous system. In the present study, 277 differentially expressed miRs were found in dioxin treated SK-N-SH cells. Among these differentially expressed miRs, 53 miRs exhibited more than 0.4-fold of changes in TCDD treated groups (0.1 nM). Other studies looking at TCDD treatments have observed even larger changes in miR expression (upwards to 2.5-fold), but they also used higher concentrations of TCDD, which could partially explain a stronger response in expression levels^20–23^. The relatively low TCDD concentration used in the present study was close to the average serum concentration of TCDD (0.01–0.1 nM) among individuals exposed to dioxins in Vietnam, Seveso Italy and Taiwan^6^. Thus, the overall changes in neuronal miR expression profiles could model environmentally relevant outcomes, which makes us more confident to further discuss the health impact association of the dioxin-induced miRs alterations.

Emerging evidence shows that miRs play important roles in human disease and cancer^30^. According to the literature, 13 miRs with more than 0.4-fold changes in the present microarray dataset are involved in neurodegenerative diseases or brain tumors, suggesting that dioxin may cause these health impacts through miRs^31–57^. Primate and human specific miRs have been considered as powerful sources for cancer biomarkers and novel therapeutic target studies^58^. Thus, primate or human-specific miRs from the present dataset might provide specific clues for further toxicological relevance analysis. The primate-specific miR, hsa-miR-608 was identified from the dataset and verified to be induced by TCDD treatment in human neuroblastoma cells. In the functional prediction of hsa-miR-608, association with glioma and/or cytoskeleton organization was found, which is consistent with the reported correlation of hsa-miR-608 with the migration of glioma stem cells^18^. Therefore, we hypothesize that glioma and/or cytoskeleton related defects might be a potential outcome of dioxin exposure in the nervous system, in which dioxin-induced dysregulation of hsa-miR-608 and/or its target genes might play roles.

CDC42, a known target gene of hse-miR-608 in a human macrophage cell line U937 and a human breast cancer cell line MT-1^19, 59^, is a small GTPase of the Rho family involved in cytoskeleton reorganization and cell migration and motility^60^. In this study, by using hsa-miR-608 mimic and inhibitor, we confirmed that hsa-miR-608 could target CDC42 and down-regulate its expression in SK-N-SH neuroblastoma cells. More importantly, we first reported that TCDD treatment could induce the expression of hsa-miR-608, which subsequently could enhance one of the post-transcriptional down-regulation processes of CDC42. However, the overall effect of TCDD treatment on CDC42 was the up-regulation of its mRNA level, suggesting that apart from the post-transcriptional down-regulation via hsa-miR-608, another up-regulatory mechanism for CDC42 expression could be involved in the dioxin effect. The presence of DRE core sequences (5′- GCGTG- 3′) in the promoter region is a common feature of classical dioxin-responsive genes^12, 61^. According to human CDC42 promoter sequence analysis (−2500 to + 50 bp)^62^, five putative DREs consensus sequences were found, suggesting a possible capability of TCDD to control CDC42 expression at the transcriptional level. This is supported by the finding that CDC42 expression was increased through over-expression of AhR in mouse Neuro2a neuroblastoma cells^63^. Therefore, we hypothesize that transcriptional up-regulation via AhR pathway might participate the dioxin-mediated CDC42 induction in the present human neuroblastoma cells. This multilevel regulation of TCDD on CDC42 expression could explain our present finding that TCDD treatment in hsa-miR-608 inhibitor transfected cells could induce CDC42 mRNA expression more obviously than that of NC transfected cells.

It is well-known that AhR mediates dioxin-induced transcriptional regulation of a series of genes. Emerging evidence has shown that these dioxin-responsive genes include not only protein-coding genes, but also non-coding genes, such as miRs^20–23^. In this study, by using the AhR antagonist, we demonstrated hsa-miR-608 as a novel dioxin-responsive non-coding gene regulated by the AhR-dependent pathway. Hsa-miR-608, located within the intron region of its host gene SEMA4G, has been hypothesized to share a common promoter with the host^64^. Through bioinformatics analysis, three DREs consensus sequences were found in the putative promoter region (chr10:100970529-100972528, the current human genome GRCh38 assembly) of hsa-miR-608, further supporting the involvement of AhR-dependent signaling in the regulation. Thus, AhR might be a communal upstream signaling molecule in the transcriptional induction and the post-transcriptional suppression of CDC42 by dioxin. In line with this notion, the alteration of CDC42 induced by TCDD was almost completely reversed by AhR angonist treatment. Moreover, the list of miRs involved in the AhR-mediated multilevel regulation of CDC42 by dioxin is still open. Based on the present miR array dataset, another up-regulated miR, has-miR-6836, is predicted to target to CDC42. It shares promoter with its host gene SNX8, which contains 10 putative consensus DRE sequences. However, the role of has-miR-6836 in dioxin-induced CDC42 dysregulation needs further investigation. Given the association of CDC42 and AhR with cytoskeleton organization^65^, this AhR mediated multilevel regulation of CDC42 by dioxin might result in interference of cytoskeleton-related cellular processes of the neurons, which merits further investigation.

In conclusion, we showed that dioxin could cause alterations in expression of a considerable fraction of miRs in human SK-N-SH neuroblastoma cells. Some differentially expressed miRs, including primate-specific hsa-miR-608, are known to be correlated with pathological conditions in the nervous system. Hsa-miR-608 was validated to be transcriptionally up-regulated upon TCDD treatment, which might be related to several cellular events in neurons, such as neuron differentiation, neuron projection, and actin cytoskeleton organization. CDC42, a cytoskeleton organization related gene, was demonstrated as a target gene of hsa-miR-608 in SK-N-SH cells, which could be significantly up-regulated upon TCDD treatment. Both transcriptional and hsa-miR-608 related post-transcriptional mechanisms are involved in the regulation of CDC42 caused by dioxin, in which AhR is an important upstream mediator.

## Materials and Methods

### Cell culture

SK-N-SH, a cell line derived from human neuroblastoma cells, was purchased from the cell resource center of the Chinese Academy of Medical Sciences (Beijing, China). Cells were maintained in Dulbecco′s modified Eagle’s medium (DMEM, Gibco, Paisley, Scotland, UK), supplemented with 10% fetal bovine serum (FBS, Corning, NY, USA), and 1% penicillin–streptomycin (Gibco, Paisley, Scotland, UK). Cells were cultured at 37 °C in a 5% CO_2_ humidified incubator.

### Exposure experiments

2,3,7,8-tetrachlorodibenzo-p-dioxin (TCDD), the most potent congener of dioxins, was purchased from Wellington Laboratories Inc. (Ontario, Canada) and dissolved in dimethyl sulfoxide (DMSO). Cells were seeded in culture dishes at an appropriate density for 24–48 hr. At 70% confluence, cells were treated with environmentally relevant low concentrations of TCDD^6^. DMSO was present at 0.1% or lower for all treatments. CH223191, an antagonist of AhR, was obtained from Sigma (St. Louis, MO, USA) and used at a concentration of 10^−6^ M. After treatments, cells were washed with phosphate-buffered saline (PBS, pH 7.4) twice and prepared for miR microarray and other experimental processes.

### MiRs isolation and miR microarray analysis

Total RNA containing miRs was extracted from SK-N-SH cells treated with 0.1% DMSO or 10^−10^ M TCDD for 24 hr. Trizol reagent (Invitrogen, Carlsbad, USA) followed by miRNeasy kit (Qiagen, Germany) were used for enrichment of miRs. This is a common method of sample processing for the miR microarray^66^. In brief, cells were lysed in Trizol/chloroform reagent according to the manufacturer’s instruction. The mixture was centrifuged at 12,000 g for 15 min at 4 °C, and the upper aqueous phase was mixed with 1.5 volume of 100% ethanol. This mixture was then processed using an miRNeasy kit according to the manufacturer’s instruction. The total RNAs of three control groups and three TCDD-treated groups were separately hybridized to Affymetrix GeneChip miR 4.0 Arrays each containing 2578 human mature miR probe sets. The arrays were scanned by means of a Affymetrix GeneChip Scanner 3000 7 G system (Affymetrix, USA). Scanned images (CEL format) were imported into Affymetrix Expression Console software (version 1.4.1; Affymetrix, USA) for normalization by Robust Multichip Average (RMA) algorithm, and the generated CHP files were imported into Affymetrix Transcriptome Analysis Console software (version 3.0; Affymetrix, USA) for further analysis. miRs expression data were statistically analysed using one-way ANOVA, and p value < 0.05 was considered as a significant change. The miRs with more than 0.4-fold of changes were further investigated.

### A review on association of candidate miRs with the nervous system

Published literature in English until February 2017 was searched for relevant studies. Articles were primarily collected from database searches in PubMed, Web of Science and Google Scholar. The search string referenced the miRs (more than 0.4-fold of changes) and neurodevelopmental abnormalities or neurodegenerative disorders (e.g., cognition, memory, neurobehavior, glioma, glioblastoma, glioblastoma multiforme, medulloblastoma, brain tumors, Alzheimer’s Disease, Parkinson Disease, Amyotrophic Lateral Sclerosis Disease, Myotonic Dystrophy Type 2 Disease, Huntington’s Disease, Neurofibromatosis Type 1 Disease). All terms were searched using both controlled vocabulary and free text words in titles and abstracts. In addition, studies that did not contain original data, such as reviews, editorials or conference abstracts, were excluded.

### Quantitative analysis of candidate miRs

MiRs from SK-N-SH cells were isolated by miRcute miR isolation kit (Tiangen Biotech, Beijing, China) according to the manufacturer’s instruction. In brief, cells were lysed with Buffer MZ/chloroform reagent (the similar function as Trizol/chloroform reagent). The mixture was centrifuged at 12,000 g for 15 min at 4 °C, and the upper aqueous phase was transferred to an absorption column in the miR extraction kit. The obtained mixture was then processed according to the manufacturer’s instruction. The extracted products were reverse transcribed to cDNA using the miRcute miR First-Strand cDNA synthesis kit (Tiangen Biotech). cDNAs were stored at −20 °C. All procedures were carried out according to the instructions provided by the manufacturer. Expression levels of the mature miRs were quantified by qPCR analysis with small nuclear RNA U6 (snRNA) as an internal reference for normalization. The qPCR analysis was performed on a LightCycler Roche 480 instrument (LC-480II, Roche, USA) using miRcute miR qPCR detection kit (SYBR Green) (Tiangen Biotech). Forward and reverse primers were provided by Tiangen Biotech. The qPCR analyses were performed under the following conditions: 94 °C 2 min, 1 cycle; 94 °C 20 s, 60 °C 34 s, 40 cycles. The data were analyzed using a ∆∆Ct method^67^.

### Bioinformatics analysis

Target genes of miRs were predicted by updated versions of TargetScan v7.0 (updated: Aug 2015, http://www.targetscan.org/vert_71/) and miRDB (updated: Mar 2015, http://www.mirdb.org/miRDB/). Communal predicted target genes of the miRs from the two tools were taken into account. The predicted target genes were then subjected to KEGG enrichment analyses and GO analysis using DAVID (the Database for Annotation, Visualization and Integrated Discovery, https://david.ncifcrf.gov/home.jsp) with the total human genome information as the background.

### Cell transfection

The cells were seeded in 12-well plates at a concentration of 2 × 10^5^ cell/well one day before transfection. The NC, hsa-miR-608 mimics or inhibitor (GenePharma, China) at 50 nM for each well were introduced into the cells using Lipofectamine RNAiMAX transfection reagent (Invitrogen) according to the manufacturer’s instruction. Twenty-four hours after transfection, cells were subjected directly to qPCR analyses or to treatment with TCDD at 10^−10^ M for 36 hr followed by the gene expression testing.

### Quantitative analysis of the selected gene

For revealing mRNA levels of the selected gene, CDC42, total RNAs were extracted from SK-N-SH cells using RNAprep pure Cell/Bacteria Kit (Tiangen Biotech) according to manufacturer’s instructions. Total RNA (2.5 µg) was reverse transcribed using Thermo Scientific RevertAid First Strand cDNA Synthesis Kit (Thermo Fisher Scientific) according to the manufacturer’s instructions. The expression level of CDC42 was quantified by qPCR using GoTaq® qPCR Master Mix kit according to the manufacturer’s instructions (Promega, Madison, WI, USA). The SYBR green signal was detected by the LightCycler 480 Instrument (LC-480II, Roche). The conditions of the qPCR were as follows: 5 min at 95 °C, followed by 45 cycles each consisting of 10 s at 95 °C, 20 s at 60 °C and 30 s at 72 °C, and a cooling step of 16 s at 42 °C. The data were analyzed using a ∆∆Ct method^67^. The sequences of mRNA primer pairs used were as follows: CDC42 (NM_001791.3), forward primer 5′-GATGGTGCTGTTGGTAAA-3′ and reverse primer 5′-TAACTCAGCGGTCGTAAT-3′; 18 S (NR_003286), forward primer 5′-CGCCGCTAGAGGTGAAATTC-3′ and reverse primer 5′-TTGGCAAATGCTTTCGCTC-3′.

### Statistical analysis

Data are expressed as mean ± S.E. from at least three independent experiments. The differences were assessed using one-way ANOVA test with Bonferroni correction using IBM SPSS19.0 software (IBM Corp., Armonk, NY) and p < 0.05 (*) or p < 0.01 (**) was considered to be statistically significant. The charts were drawn by Origin 9.0 software (OriginLab, MA).

## Electronic supplementary material

Supplementary Information
