# Supplementary material for: CDC42 expression is altered by dioxin exposure and mediated by multilevel regulations via AhR in human neuroblastoma cells
Source: Sci Rep. 2017 Aug 31;7:10103. doi: 10.1038/s41598-017-10311-3 (PMC5578991; doi:10.1038/s41598-017-10311-3)
Supplement: Supplementary file 1 — Supplementary Information [file 41598_2017_10311_MOESM1_ESM.pdf]

Supplementary information

CDC42 expression is altered by dioxin exposure and mediated by multilevel regulations via AhR in human neuroblastoma cells

Tuan Xu[1, 2, +], Heidi Q. Xie[1, 2, +], Yunping Li[1, 2], Yingjie Xia[1, 2], Yangsheng Chen[1, 2], Li Xu[1, 2], Lingyun Wang[1,2], Bin Zhao[1, 2, *]

[1] State Key Laboratory of Environmental Chemistry and Ecotoxicology, Research Center for Eco-Environmental Sciences, Chinese Academy of Sciences, Beijing, 100085, China.

[2] University of Chinese Academy of Sciences, Beijing, 100085, China.

[+] These authors contributed equally to this work.

*Corresponding author email: binzhao@rcees.ac.cn

Supplementary table 1: A list of differentially expressed miRs from the microarray analysis

miRBase: Accession	miR	Fold-Change	p-value	Description
MIMAT0028115	hsa-miR-7109-5p	3.60	1.51E-02	TCDD up vs DMSO
MIMAT0004591	hsa-miR-124-5p	3.10	2.20E-03	TCDD up vs DMSO
MIMAT0003340	hsa-miR-542-5p	2.34	1.02E-02	TCDD up vs DMSO
MIMAT0019743	hsa-miR-4667-5p	2.07	6.47E-03	TCDD up vs DMSO
MIMAT0027460	hsa-miR-6780a-5p	1.91	3.49E-02	TCDD up vs DMSO
MIMAT0017984	hsa-miR-3607-5p	1.90	4.64E-02	TCDD up vs DMSO
MIMAT0002809	hsa-miR-146b-5p	1.90	1.00E-02	TCDD up vs DMSO
MIMAT0020925	hsa-miR-550a-3-5p	1.87	2.72E-02	TCDD up vs DMSO
MIMAT0022695	hsa-miR-212-5p	1.77	4.55E-02	TCDD up vs DMSO
MIMAT0005896	hsa-miR-1244	1.73	2.27E-02	TCDD up vs DMSO
MIMAT0027481	hsa-miR-6790-3p	1.63	4.48E-02	TCDD up vs DMSO
MIMAT0015019	hsa-miR-3147	1.59	1.75E-02	TCDD up vs DMSO
MIMAT0007882	hsa-miR-1909-5p	1.57	3.30E-02	TCDD up vs DMSO
MIMAT0003337	hsa-miR-659-3p	1.54	2.31E-02	TCDD up vs DMSO
MIMAT0002177	hsa-miR-486-5p	1.53	8.07E-03	TCDD up vs DMSO
MIMAT0004762	hsa-miR-486-3p	1.53	8.07E-03	TCDD up vs DMSO
MIMAT0018967	hsa-miR-4448	1.52	1.27E-02	TCDD up vs DMSO
MIMAT0000459	hsa-miR-193a-3p	1.51	2.66E-02	TCDD up vs DMSO
MIMAT0027383	hsa-miR-6741-5p	1.51	3.45E-02	TCDD up vs DMSO
MIMAT0028116	hsa-miR-7109-3p	1.47	8.00E-03	TCDD up vs DMSO
MIMAT0019711	hsa-miR-4649-5p	1.47	3.80E-02	TCDD up vs DMSO
MIMAT0017352	hsa-miR-2277-5p	1.47	8.89E-03	TCDD up vs DMSO
MIMAT0027574	hsa-miR-6836-5p	1.45	3.03E-03	TCDD up vs DMSO
MIMAT0030017	hsa-miR-7702	1.45	1.65E-02	TCDD up vs DMSO
MIMAT0018005	hsa-miR-3622b-5p	1.44	9.84E-04	TCDD up vs DMSO
MIMAT0018006	hsa-miR-3622b-3p	1.44	9.84E-04	TCDD up vs DMSO
MIMAT0026618	hsa-miR-585-5p	1.43	3.71E-02	TCDD up vs DMSO
MIMAT0000261	hsa-miR-183-5p	1.42	2.84E-03	TCDD up vs DMSO
MIMAT0016849	hsa-miR-4294	1.42	4.83E-02	TCDD up vs DMSO
MIMAT0003276	hsa-miR-608	1.41	2.41E-02	TCDD up vs DMSO
MIMAT0014990	hsa-miR-3127-5p	1.41	1.33E-02	TCDD up vs DMSO
MIMAT0019201	hsa-miR-3127-3p	1.41	1.33E-02	TCDD up vs DMSO
MIMAT0016889	hsa-miR-4327	1.40	3.05E-02	TCDD up vs DMSO
MIMAT0017988	hsa-miR-3611	1.39	4.44E-02	TCDD up vs DMSO
MIMAT0019755	hsa-miR-4673	1.38	4.66E-02	TCDD up vs DMSO
MIMAT0016868	hsa-miR-4314	1.38	6.61E-03	TCDD up vs DMSO
MIMAT0022696	hsa-miR-301a-5p	1.37	1.76E-02	TCDD up vs DMSO
MIMAT0000688	hsa-miR-301a-3p	1.37	1.76E-02	TCDD up vs DMSO
MIMAT0002806	hsa-miR-490-3p	1.37	8.77E-03	TCDD up vs DMSO
MIMAT0004804	hsa-miR-615-5p	1.36	4.71E-02	TCDD up vs DMSO

MIMAT0027547	hsa-miR-6823-3p	1.36	3.52E-02	TCDD up vs DMSO
MIMAT0019829	hsa-miR-4717-5p	1.35	3.75E-02	TCDD up vs DMSO
MIMAT0019830	hsa-miR-4717-3p	1.35	3.75E-02	TCDD up vs DMSO
MIMAT0019066	hsa-miR-4527	1.35	6.88E-03	TCDD up vs DMSO
MIMAT0018112	hsa-miR-3684	1.35	7.90E-03	TCDD up vs DMSO
MIMAT0027652	hsa-miR-6876-5p	1.34	3.00E-03	TCDD up vs DMSO
MIMAT0001339	hsa-miR-422a	1.34	1.74E-03	TCDD up vs DMSO
MIMAT0005902	hsa-miR-1250-5p	1.32	1.32E-02	TCDD up vs DMSO
MIMAT0015076	hsa-miR-3192-5p	1.32	1.07E-02	TCDD up vs DMSO
MIMAT0003279	hsa-miR-611	1.31	4.47E-02	TCDD up vs DMSO
MIMAT0027380	hsa-miR-6739-3p	1.31	1.12E-02	TCDD up vs DMSO
MIMAT0000684	hsa-miR-302a-3p	1.31	1.43E-02	TCDD up vs DMSO
MIMAT0027432	hsa-miR-6766-5p	1.31	5.47E-03	TCDD up vs DMSO
MIMAT0007889	hsa-miR-1914-5p	1.30	3.93E-02	TCDD up vs DMSO
MIMAT0019903	hsa-miR-4758-5p	1.30	1.39E-02	TCDD up vs DMSO
MIMAT0019904	hsa-miR-4758-3p	1.30	1.39E-02	TCDD up vs DMSO
MIMAT0028111	hsa-miR-7107-5p	1.28	1.09E-03	TCDD up vs DMSO
MIMAT0028112	hsa-miR-7107-3p	1.28	1.09E-03	TCDD up vs DMSO
MIMAT0019023	hsa-miR-4489	1.27	2.64E-02	TCDD up vs DMSO
MIMAT0019206	hsa-miR-3150a-5p	1.27	1.39E-02	TCDD up vs DMSO
MIMAT0015023	hsa-miR-3150a-3p	1.27	1.39E-02	TCDD up vs DMSO
MIMAT0027381	hsa-miR-6740-5p	1.27	4.06E-02	TCDD up vs DMSO
MIMAT0004564	hsa-miR-214-5p	1.27	7.13E-03	TCDD up vs DMSO
MIMAT0027358	hsa-miR-6728-3p	1.27	2.24E-02	TCDD up vs DMSO
MIMAT0016855	hsa-miR-4302	1.27	1.80E-02	TCDD up vs DMSO
MIMAT0027444	hsa-miR-6772-5p	1.26	3.93E-02	TCDD up vs DMSO
MIMAT0009199	hsa-miR-365a-5p	1.26	2.22E-02	TCDD up vs DMSO
MIMAT0000710	hsa-miR-365a-3p	1.26	2.22E-02	TCDD up vs DMSO
MIMAT0011162	hsa-miR-2117	1.25	3.99E-02	TCDD up vs DMSO
MIMAT0030415	hsa-miR-1273h-5p	1.25	3.48E-02	TCDD up vs DMSO
MIMAT0030416	hsa-miR-1273h-3p	1.25	3.48E-02	TCDD up vs DMSO
MIMAT0019014	hsa-miR-4480	1.25	2.18E-03	TCDD up vs DMSO
MIMAT0018178	hsa-miR-3180-4	1.24	3.32E-02	TCDD up vs DMSO
MIMAT0002880	hsa-miR-508-3p	1.24	2.39E-02	TCDD up vs DMSO
MIMAT0019058	hsa-miR-4521	1.24	3.81E-02	TCDD up vs DMSO
MIMAT0002852	hsa-miR-517a-3p	1.23	2.35E-02	TCDD up vs DMSO
MIMAT0002857	hsa-miR-517b-3p	1.23	2.35E-02	TCDD up vs DMSO
MIMAT0004780	hsa-miR-532-3p	1.23	7.80E-03	TCDD up vs DMSO
MIMAT0015064	hsa-miR-3184-5p	1.23	3.86E-02	TCDD up vs DMSO
MIMAT0022731	hsa-miR-3184-3p	1.23	3.86E-02	TCDD up vs DMSO
MIMAT0022476	hsa-miR-5692c	1.23	4.08E-02	TCDD up vs DMSO
MIMAT0004901	hsa-miR-298	1.23	3.69E-02	TCDD up vs DMSO
MIMAT0026619	hsa-miR-597-3p	1.22	2.67E-02	TCDD up vs DMSO

MIMAT0021085	hsa-miR-5093	1.21	1.54E-02	TCDD up vs DMSO
MIMAT0030018	hsa-miR-7703	1.21	4.26E-02	TCDD up vs DMSO
MIMAT0019971	hsa-miR-4796-3p	1.21	4.01E-02	TCDD up vs DMSO
MIMAT0019722	hsa-miR-4655-3p	1.21	2.59E-02	TCDD up vs DMSO
MIMAT0019862	hsa-miR-4736	1.21	3.89E-02	TCDD up vs DMSO
MIMAT0016903	hsa-miR-4273	1.20	2.01E-02	TCDD up vs DMSO
MIMAT0015030	hsa-miR-3156-5p	1.20	1.65E-02	TCDD up vs DMSO
MIMAT0019209	hsa-miR-3156-3p	1.20	1.65E-02	TCDD up vs DMSO
MIMAT0021019	hsa-miR-5000-5p	1.19	1.65E-02	TCDD up vs DMSO
MIMAT0021020	hsa-miR-5000-3p	1.19	1.65E-02	TCDD up vs DMSO
MIMAT0004588	hsa-miR-27b-5p	1.19	3.04E-02	TCDD up vs DMSO
MIMAT0000419	hsa-miR-27b-3p	1.19	3.04E-02	TCDD up vs DMSO
MIMAT0016856	hsa-miR-4303	1.18	1.50E-02	TCDD up vs DMSO
MIMAT0004694	hsa-miR-342-5p	1.18	4.21E-02	TCDD up vs DMSO
MIMAT0005449	hsa-miR-523-5p	1.17	4.90E-02	TCDD up vs DMSO
MIMAT0002840	hsa-miR-523-3p	1.17	4.90E-02	TCDD up vs DMSO
MIMAT0019977	hsa-miR-4799-3p	1.17	2.41E-02	TCDD up vs DMSO
MIMAT0019819	hsa-miR-4712-3p	1.17	2.71E-02	TCDD up vs DMSO
MIMAT0002808	hsa-miR-511-5p	1.17	2.30E-02	TCDD up vs DMSO
MIMAT0018114	hsa-miR-3686	1.17	1.44E-02	TCDD up vs DMSO
MIMAT0006789	hsa-miR-1468-5p	1.16	2.16E-02	TCDD up vs DMSO
MIMAT0018353	hsa-miR-3938	1.16	4.98E-04	TCDD up vs DMSO
MIMAT0019063	hsa-miR-4524a-3p	1.16	1.50E-03	TCDD up vs DMSO
MIMAT0000266	hsa-miR-205-5p	1.15	3.50E-02	TCDD up vs DMSO
MIMAT0009197	hsa-miR-205-3p	1.15	3.50E-02	TCDD up vs DMSO
MIMAT0028217	hsa-miR-7153-3p	1.15	4.47E-02	TCDD up vs DMSO
MIMAT0000714	hsa-miR-302b-5p	1.15	2.14E-02	TCDD up vs DMSO
MIMAT0000715	hsa-miR-302b-3p	1.15	2.14E-02	TCDD up vs DMSO
MIMAT0016916	hsa-miR-4286	1.14	2.81E-06	TCDD up vs DMSO
MIMAT0027373	hsa-miR-6736-5p	1.14	3.95E-02	TCDD up vs DMSO
MIMAT0009979	hsa-miR-2054	1.13	2.66E-02	TCDD up vs DMSO
MIMAT0014989	hsa-miR-3126-5p	1.13	4.02E-02	TCDD up vs DMSO
MIMAT0015377	hsa-miR-3126-3p	1.13	4.02E-02	TCDD up vs DMSO
MIMAT0003295	hsa-miR-626	1.12	3.49E-02	TCDD up vs DMSO
MIMAT0018070	hsa-miR-3650	1.12	3.23E-04	TCDD up vs DMSO
MIMAT0021082	hsa-miR-5090	1.11	1.07E-02	TCDD up vs DMSO
MIMAT0022260	hsa-miR-5572	1.10	2.71E-03	TCDD up vs DMSO
MIMAT0022299	hsa-miR-5590-5p	1.10	2.72E-02	TCDD up vs DMSO
MIMAT0004805	hsa-miR-616-3p	1.10	1.99E-02	TCDD up vs DMSO
MIMAT0004748	hsa-miR-423-5p	1.09	4.75E-02	TCDD up vs DMSO
MIMAT0001340	hsa-miR-423-3p	1.09	4.75E-02	TCDD up vs DMSO
MIMAT0000276	hsa-miR-219a-5p	1.08	7.34E-04	TCDD up vs DMSO
MIMAT0019005	hsa-miR-4477b	1.06	2.80E-02	TCDD up vs DMSO

MIMAT0000063	hsa-let-7b-5p	1.05	3.74E-02	TCDD up vs DMSO
MIMAT0018443	hsa-miR-374c-5p	1.04	1.92E-02	TCDD up vs DMSO
MIMAT0022735	hsa-miR-374c-3p	1.04	1.92E-02	TCDD up vs DMSO
MIMAT0019758	hsa-miR-4676-5p	-1.08	3.28E-02	TCDD down vs DMSO
MIMAT0019759	hsa-miR-4676-3p	-1.08	3.28E-02	TCDD down vs DMSO
MIMAT0019028	hsa-miR-4493	-1.08	3.88E-02	TCDD down vs DMSO
MIMAT0018981	hsa-miR-4459	-1.09	4.14E-02	TCDD down vs DMSO
MIMAT0014987	hsa-miR-548s	-1.10	1.97E-02	TCDD down vs DMSO
MIMAT0003316	hsa-miR-646	-1.10	4.03E-02	TCDD down vs DMSO
MIMAT0000098	hsa-miR-100-5p	-1.11	3.11E-02	TCDD down vs DMSO
MIMAT0004512	hsa-miR-100-3p	-1.11	3.11E-02	TCDD down vs DMSO
MIMAT0027650	hsa-miR-6875-5p	-1.11	4.74E-02	TCDD down vs DMSO
MIMAT0014978	hsa-miR-3116	-1.11	3.83E-02	TCDD down vs DMSO
MIMAT0023712	hsa-miR-6087	-1.11	2.06E-02	TCDD down vs DMSO
MIMAT0018925	hsa-miR-1268b	-1.11	4.95E-02	TCDD down vs DMSO
MIMAT0003226	hsa-miR-562	-1.11	8.72E-03	TCDD down vs DMSO
MIMAT0019956	hsa-miR-4787-5p	-1.12	3.51E-02	TCDD down vs DMSO
MIMAT0005918	hsa-miR-1265	-1.12	4.85E-02	TCDD down vs DMSO
MIMAT0011156	hsa-miR-2114-5p	-1.12	4.66E-02	TCDD down vs DMSO
MIMAT0007885	hsa-miR-1911-5p	-1.12	1.95E-03	TCDD down vs DMSO
MIMAT0001631	hsa-miR-451a	-1.14	3.13E-03	TCDD down vs DMSO
MIMAT0003329	hsa-miR-411-5p	-1.14	3.31E-02	TCDD down vs DMSO
MIMAT0004813	hsa-miR-411-3p	-1.14	3.31E-02	TCDD down vs DMSO
MIMAT0025844	hsa-miR-6716-5p	-1.14	4.53E-02	TCDD down vs DMSO
MIMAT0025845	hsa-miR-6716-3p	-1.14	4.53E-02	TCDD down vs DMSO
MIMAT0004701	hsa-miR-338-5p	-1.14	2.58E-02	TCDD down vs DMSO
MIMAT0000763	hsa-miR-338-3p	-1.14	2.58E-02	TCDD down vs DMSO
MIMAT0016904	hsa-miR-4276	-1.14	4.95E-02	TCDD down vs DMSO
MIMAT0027374	hsa-miR-6736-3p	-1.15	4.34E-02	TCDD down vs DMSO
MIMAT0028113	hsa-miR-7108-5p	-1.15	3.44E-02	TCDD down vs DMSO
MIMAT0010313	hsa-miR-762	-1.16	1.44E-02	TCDD down vs DMSO
MIMAT0019778	hsa-miR-4689	-1.16	1.82E-02	TCDD down vs DMSO
MIMAT0028230	hsa-miR-7160-5p	-1.16	1.56E-03	TCDD down vs DMSO
MIMAT0028231	hsa-miR-7160-3p	-1.16	1.56E-03	TCDD down vs DMSO
MIMAT0001532	hsa-miR-448	-1.17	2.43E-02	TCDD down vs DMSO
MIMAT0019841	hsa-miR-4724-5p	-1.17	2.20E-02	TCDD down vs DMSO
MIMAT0019842	hsa-miR-4724-3p	-1.17	2.20E-02	TCDD down vs DMSO
MIMAT0030424	hsa-miR-7849-3p	-1.17	3.81E-02	TCDD down vs DMSO
MIMAT0024598	hsa-miR-6125	-1.17	3.99E-02	TCDD down vs DMSO
MIMAT0015022	hsa-miR-3149	-1.17	4.20E-02	TCDD down vs DMSO
MIMAT0022491	hsa-miR-5698	-1.17	2.30E-02	TCDD down vs DMSO
MIMAT0004503	hsa-miR-29a-5p	-1.17	3.38E-02	TCDD down vs DMSO
MIMAT0000086	hsa-miR-29a-3p	-1.17	3.38E-02	TCDD down vs DMSO

MIMAT0019729	hsa-miR-4661-5p	-1.18	3.73E-02	TCDD down vs DMSO
MIMAT0019730	hsa-miR-4661-3p	-1.18	3.73E-02	TCDD down vs DMSO
MIMAT0027387	hsa-miR-6743-5p	-1.18	4.67E-02	TCDD down vs DMSO
MIMAT0024613	hsa-miR-6129	-1.18	3.47E-02	TCDD down vs DMSO
MIMAT0019948	hsa-miR-4784	-1.18	4.86E-02	TCDD down vs DMSO
MIMAT0019944	hsa-miR-4782-5p	-1.18	2.58E-02	TCDD down vs DMSO
MIMAT0019945	hsa-miR-4782-3p	-1.18	2.58E-02	TCDD down vs DMSO
MIMAT0007347	hsa-miR-1469	-1.19	5.04E-03	TCDD down vs DMSO
MIMAT0019229	hsa-miR-3940-5p	-1.19	2.61E-02	TCDD down vs DMSO
MIMAT0019766	hsa-miR-4681	-1.19	2.81E-03	TCDD down vs DMSO
MIMAT0016915	hsa-miR-4284	-1.19	3.74E-02	TCDD down vs DMSO
MIMAT0024782	hsa-miR-6165	-1.19	4.41E-02	TCDD down vs DMSO
MIMAT0005911	hsa-miR-1260a	-1.19	4.59E-03	TCDD down vs DMSO
MIMAT0018989	hsa-miR-548ai	-1.19	2.13E-02	TCDD down vs DMSO
MIMAT0005955	hsa-miR-1197	-1.20	2.06E-02	TCDD down vs DMSO
MIMAT0028219	hsa-miR-7154-3p	-1.20	1.19E-02	TCDD down vs DMSO
MIMAT0005829	hsa-miR-1184	-1.20	3.32E-02	TCDD down vs DMSO
MIMAT0005885	hsa-miR-1295a	-1.20	2.14E-02	TCDD down vs DMSO
MIMAT0005950	hsa-miR-1306-3p	-1.21	3.64E-02	TCDD down vs DMSO
MIMAT0017992	hsa-miR-3614-5p	-1.21	2.08E-03	TCDD down vs DMSO
MIMAT0017993	hsa-miR-3614-3p	-1.21	2.08E-03	TCDD down vs DMSO
MIMAT0019062	hsa-miR-4524a-5p	-1.21	4.64E-02	TCDD down vs DMSO
MIMAT0027648	hsa-miR-6874-5p	-1.21	1.76E-03	TCDD down vs DMSO
MIMAT0027359	hsa-miR-6729-5p	-1.21	1.29E-02	TCDD down vs DMSO
MIMAT0022923	hsa-miR-376b-5p	-1.21	3.75E-02	TCDD down vs DMSO
MIMAT0002172	hsa-miR-376b-3p	-1.21	3.75E-02	TCDD down vs DMSO
MIMAT0005919	hsa-miR-548o-3p	-1.22	2.07E-02	TCDD down vs DMSO
MIMAT0000724	hsa-miR-372-3p	-1.22	3.44E-02	TCDD down vs DMSO
MIMAT0001536	hsa-miR-429	-1.23	3.04E-02	TCDD down vs DMSO
MIMAT0003150	hsa-miR-455-5p	-1.23	4.81E-02	TCDD down vs DMSO
MIMAT0004784	hsa-miR-455-3p	-1.23	4.81E-02	TCDD down vs DMSO
MIMAT0019712	hsa-miR-4649-3p	-1.23	1.59E-02	TCDD down vs DMSO
MIMAT0019874	hsa-miR-4743-5p	-1.24	1.47E-02	TCDD down vs DMSO
MIMAT0022978	hsa-miR-4743-3p	-1.24	1.47E-02	TCDD down vs DMSO
MIMAT0027602	hsa-miR-6851-5p	-1.24	2.20E-02	TCDD down vs DMSO
MIMAT0027603	hsa-miR-6851-3p	-1.24	2.20E-02	TCDD down vs DMSO
MIMAT0011157	hsa-miR-2114-3p	-1.24	2.80E-02	TCDD down vs DMSO
MIMAT0019942	hsa-miR-4781-5p	-1.24	1.70E-02	TCDD down vs DMSO
MIMAT0021084	hsa-miR-5092	-1.25	1.26E-02	TCDD down vs DMSO
MIMAT0018958	hsa-miR-4440	-1.25	2.46E-02	TCDD down vs DMSO
MIMAT0028119	hsa-miR-7111-5p	-1.25	4.94E-02	TCDD down vs DMSO
MIMAT0028120	hsa-miR-7111-3p	-1.25	4.94E-02	TCDD down vs DMSO
MIMAT0027365	hsa-miR-6732-5p	-1.25	3.92E-02	TCDD down vs DMSO

MIMAT0019796	hsa-miR-4700-5p	-1.26	1.27E-02	TCDD down vs DMSO
MIMAT0014998	hsa-miR-3133	-1.26	3.77E-02	TCDD down vs DMSO
MIMAT0000448	hsa-miR-136-5p	-1.26	3.13E-02	TCDD down vs DMSO
MIMAT0004606	hsa-miR-136-3p	-1.26	3.13E-02	TCDD down vs DMSO
MIMAT0027412	hsa-miR-6756-5p	-1.27	4.23E-02	TCDD down vs DMSO
MIMAT0027413	hsa-miR-6756-3p	-1.27	4.23E-02	TCDD down vs DMSO
MIMAT0015031	hsa-miR-3157-5p	-1.27	4.05E-02	TCDD down vs DMSO
MIMAT0019210	hsa-miR-3157-3p	-1.27	4.05E-02	TCDD down vs DMSO
MIMAT0030426	hsa-miR-7851-3p	-1.27	4.91E-02	TCDD down vs DMSO
MIMAT0005452	hsa-miR-519a-5p	-1.27	1.21E-03	TCDD down vs DMSO
MIMAT0002869	hsa-miR-519a-3p	-1.27	1.21E-03	TCDD down vs DMSO
MIMAT0015040	hsa-miR-3166	-1.28	2.99E-02	TCDD down vs DMSO
MIMAT0027659	hsa-miR-6879-3p	-1.28	4.46E-02	TCDD down vs DMSO
MIMAT0018189	hsa-miR-3915	-1.28	2.31E-03	TCDD down vs DMSO
MIMAT0018351	hsa-miR-3936	-1.28	1.77E-02	TCDD down vs DMSO
MIMAT0016847	hsa-miR-378c	-1.28	2.71E-02	TCDD down vs DMSO
MIMAT0004971	hsa-miR-921	-1.28	4.36E-02	TCDD down vs DMSO
MIMAT0019198	hsa-miR-3120-5p	-1.28	3.62E-02	TCDD down vs DMSO
MIMAT0014982	hsa-miR-3120-3p	-1.28	3.62E-02	TCDD down vs DMSO
MIMAT0026555	hsa-miR-329-5p	-1.29	6.87E-03	TCDD down vs DMSO
MIMAT0001629	hsa-miR-329-3p	-1.29	6.87E-03	TCDD down vs DMSO
MIMAT0004909	hsa-miR-450b-5p	-1.29	4.31E-02	TCDD down vs DMSO
MIMAT0018179	hsa-miR-3907	-1.30	2.22E-02	TCDD down vs DMSO
MIMAT0019002	hsa-miR-4475	-1.30	2.25E-02	TCDD down vs DMSO
MIMAT0016865	hsa-miR-4313	-1.31	4.82E-02	TCDD down vs DMSO
MIMAT0019025	hsa-miR-4490	-1.32	3.83E-02	TCDD down vs DMSO
MIMAT0016918	hsa-miR-4288	-1.32	2.57E-02	TCDD down vs DMSO
MIMAT0019060	hsa-miR-4522	-1.32	1.43E-02	TCDD down vs DMSO
MIMAT0005881	hsa-miR-1291	-1.33	1.63E-03	TCDD down vs DMSO
MIMAT0027640	hsa-miR-6870-5p	-1.33	2.54E-02	TCDD down vs DMSO
MIMAT0027641	hsa-miR-6870-3p	-1.33	2.54E-02	TCDD down vs DMSO
MIMAT0019211	hsa-miR-3158-5p	-1.34	3.29E-02	TCDD down vs DMSO
MIMAT0015032	hsa-miR-3158-3p	-1.34	3.29E-02	TCDD down vs DMSO
MIMAT0015088	hsa-miR-514b-3p	-1.34	3.96E-02	TCDD down vs DMSO
MIMAT0005823	hsa-miR-1178-3p	-1.34	2.83E-03	TCDD down vs DMSO
MIMAT0002845	hsa-miR-526a	-1.34	2.36E-02	TCDD down vs DMSO
MIMAT0019010	hsa-miR-3689f	-1.37	1.55E-02	TCDD down vs DMSO
MIMAT0015081	hsa-miR-548x-3p	-1.37	2.57E-04	TCDD down vs DMSO
MIMAT0032115	hsa-miR-548ae-5p	-1.37	4.77E-02	TCDD down vs DMSO
MIMAT0018954	hsa-miR-548ae-3p	-1.37	4.77E-02	TCDD down vs DMSO
MIMAT0005795	hsa-miR-1323	-1.38	1.30E-02	TCDD down vs DMSO
MIMAT0019981	hsa-miR-4802-5p	-1.38	2.89E-02	TCDD down vs DMSO
MIMAT0018119	hsa-miR-3690	-1.39	1.18E-02	TCDD down vs DMSO

MIMAT0015018	hsa-miR-3146	-1.40	1.23E-02	TCDD down vs DMSO
MIMAT0015010	hsa-miR-3141	-1.40	3.99E-02	TCDD down vs DMSO
MIMAT0019887	hsa-miR-4750-5p	-1.40	4.77E-02	TCDD down vs DMSO
MIMAT0019007	hsa-miR-3689c	-1.40	1.69E-03	TCDD down vs DMSO
MIMAT0018990	hsa-miR-548aj-3p	-1.41	8.33E-03	TCDD down vs DMSO
MIMAT0027684	hsa-miR-6892-5p	-1.41	1.07E-02	TCDD down vs DMSO
MIMAT0027685	hsa-miR-6892-3p	-1.41	1.07E-02	TCDD down vs DMSO
MIMAT0027592	hsa-miR-6846-5p	-1.43	3.32E-02	TCDD down vs DMSO
MIMAT0027593	hsa-miR-6846-3p	-1.43	3.32E-02	TCDD down vs DMSO
MIMAT0019805	hsa-miR-4705	-1.43	3.35E-02	TCDD down vs DMSO
MIMAT0027595	hsa-miR-6847-3p	-1.49	2.95E-02	TCDD down vs DMSO
MIMAT0019042	hsa-miR-4506	-1.49	3.60E-02	TCDD down vs DMSO
MIMAT0027366	hsa-miR-6732-3p	-1.52	4.79E-03	TCDD down vs DMSO
MIMAT0000756	hsa-miR-326	-1.53	8.87E-03	TCDD down vs DMSO
MIMAT0019026	hsa-miR-4491	-1.56	2.76E-02	TCDD down vs DMSO
MIMAT0027682	hsa-miR-6891-5p	-1.64	3.25E-02	TCDD down vs DMSO
MIMAT0025468	hsa-miR-6506-5p	-1.66	1.04E-02	TCDD down vs DMSO
MIMAT0006790	hsa-miR-675-3p	-1.67	3.80E-02	TCDD down vs DMSO
MIMAT0025472	hsa-miR-6508-5p	-1.75	3.47E-02	TCDD down vs DMSO
MIMAT0027538	hsa-miR-6819-5p	-1.83	2.23E-02	TCDD down vs DMSO
MIMAT0027539	hsa-miR-6819-3p	-1.83	2.23E-02	TCDD down vs DMSO
MIMAT0016862	hsa-miR-4310	-2.88	2.74E-02	TCDD down vs DMSO

Supplementary table 2: A list of reported target genes of hsa-miR-608

Gene ID	Symbol	Description	Cell/tissue	Reference
6469	SHH	sonic hedgehog	Human chondrocytes	1
595	CCND1	cyclin D1	A549 tumor cells (human alveolar basal epithelial cells)	2
1727	CYB5R3	cytochrome b5 reductase 3		
5296	PIK3R2	phosphoinositide-3-kinase regulatory subunit 2		
6667	SP1	Sp1 transcription factor		
207	AKT1	AKT serine/threonine kinase 1		
43	ACHE	acetylcholinesterase (Cartwright blood group)	Human U937 cells and HEK-293T cell lines	3
960	CD44	CD44 molecule (Indian blood group)	Human breast cancer cell lines	4
998	CDC42	cell division cycle 42	Human breast cancer cell lines	4
			Human U937 cells and HEK-293T cell lines	3
1289	COL5A1	collagen type V alpha 1 chain	HT1080 cell lines	5
3569	IL6	interleukin 6	Human U937 cells and	3

			HEK-293T cell lines	
			HEK293 and Hela cell lines	6
			HEK293 and Hela cell lines	7
4282	MIF	macrophage migration inhibitory factor (glycosylation-inhibiting factor)	Human hepatocellular carcinoma	8
			Human glioma stem cells	9
8260	NAA10	N(alpha)-acetyltransferase 10, NatA catalytic subunit	Human SW480 and SW620 cell lines	10
599	BCL2L2	BCL2 like 2	Human chordoma cell	11
598	BCL2L1	BCL2 like 1	Human chordoma cell	11
			A549 tumor cells	2
1956	EGFR	epidermal growth factor receptor	Human chordoma cell lines	11
4233	MET	MET proto-oncogene, receptor tyrosine kinase		

Reference

1 Akhtar, N., Makki, M. S. & Haqqi, T. M. MicroRNA-602 and microRNA-608 regulate sonic hedgehog expression via target sites in the coding region in human chondrocytes. *Arthritis & rheumatology* **67**, 423-434, doi:10.1002/art.38952 (2015).

2 Choi, Y. C. *et al.* MicroRNA library screening identifies growth-suppressive microRNAs that regulate genes involved in cell cycle progression and apoptosis. *Experimental cell research*, doi:10.1016/j.yexcr.2015.10.012 (2015).

3 Hanin, G. *et al.* Competing targets of microRNA-608 affect anxiety and hypertension. *Human molecular genetics* **23**, 4569-4580, doi:10.1093/hmg/ddu170 (2014).

4 Jeyapalan, Z. *et al.* Expression of CD44 3'-untranslated region regulates endogenous microRNA functions in tumorigenesis and angiogenesis. *Nucleic Acids Res* **39**, 3026-3041, doi:10.1093/nar/gkq1003 (2011).

5 Laguette, M. J., Abrahams, Y., Prince, S. & Collins, M. Sequence variants within the 3'-UTR of the COL5A1 gene alters mRNA stability: implications for musculoskeletal soft tissue injuries. *Matrix biology : journal of the International Society for Matrix Biology* **30**, 338-345, doi:10.1016/j.matbio.2011.05.001 (2011).

6 Kang, J. G. *et al.* Kaposi's sarcoma-associated herpesviral IL-6 and human IL-6 open reading frames contain miRNA binding sites and are subject to cellular miRNA regulation. *The Journal of pathology* **225**, 378-389, doi:10.1002/path.2962 (2011).

7 Kang, J. G. *et al.* Kaposi's sarcoma-associated herpesvirus ORF57 promotes escape of viral and human interleukin-6 from microRNA-mediated suppression. *Journal of virology* **85**, 2620-2630, doi:10.1128/JVI.02144-10 (2011).

8 Wang, K., Liang, Q., Wei, L., Zhang, W. & Zhu, P. MicroRNA-608 acts as a prognostic marker and inhibits the cell proliferation in hepatocellular carcinoma by macrophage migration inhibitory factor. *Tumour biology : the journal of the International Society for Oncodevelopmental Biology and Medicine*, doi:10.1007/s13277-015-4213-5 (2015).

9 Wang, Z., Xue, Y., Wang, P., Zhu, J. & Ma, J. MiR-608 inhibits the migration and

invasion of glioma stem cells by targeting macrophage migration inhibitory factor. *Oncology reports* **35**, 2733-2742, doi:10.3892/or.2016.4652 (2016).

10 Yang, H. *et al.* microRNA-342-5p and miR-608 inhibit colon cancer tumorigenesis by targeting NAA10. *Oncotarget* **7**, 2709-2720, doi:10.18632/oncotarget.6458 (2016).

11 Zhang, Y., Schiff, D., Park, D. & Abounader, R. MicroRNA-608 and microRNA-34a regulate chordoma malignancy by targeting EGFR, Bcl-xL and MET. *PloS one* **9**, e91546, doi:10.1371/journal.pone.0091546 (2014).

Supplementary table 3: A list of 557 predicted target genes of hsa-miR-608

Gene ID	Symbol	description
51	ACOX1	acyl-CoA oxidase 1, palmitoyl
60	ACTB	actin, beta
91	ACVR1B	activin A receptor type IB
105	ADARB2	adenosine deaminase, RNA-specific, B2 (inactive)
117	ADCYAP1R1	adenylate cyclase activating polypeptide 1 (pituitary) receptor type I
164	AP1G1	adaptor-related protein complex 1, gamma 1 subunit
402	ARL2	ADP ribosylation factor like GTPase 2
408	ARRB1	arrestin, beta 1
477	ATP1A2	ATPase, Na+/K+ transporting, alpha 2 polypeptide
535	ATP6V0A1	ATPase, H+ transporting, lysosomal V0 subunit a1
558	AXL	AXL receptor tyrosine kinase
578	BAK1	BCL2-antagonist/killer 1
598	BCL2L1	BCL2-like 1
613	BCR	breakpoint cluster region
677	ZFP36L1	ZFP36 ring finger protein-like 1
728	C5AR1	complement component 5a receptor 1
747	DAGLA	diacylglycerol lipase, alpha
775	CACNA1C	calcium channel, voltage-dependent, L type, alpha 1C subunit
794	CALB2	calbindin 2
815	CAMK2A	calcium/calmodulin-dependent protein kinase II alpha
827	CAPN6	calpain 6
916	CD3E	CD3e molecule, epsilon (CD3-TCR complex)
920	CD4	CD4 molecule
933	CD22	CD22 molecule
954	ENTPD2	ectonucleoside triphosphate diphosphohydrolase 2
1026	CDKN1A	cyclin-dependent kinase inhibitor 1A (p21, Cip1)
1030	CDKN2B	cyclin-dependent kinase inhibitor 2B (p15, inhibits CDK4)
1041	CDSN	corneodesmosin
1050	CEBPA	CCAAT/enhancer binding protein (C/EBP), alpha
1072	CFL1	cofilin 1 (non-muscle)
1158	CKM	creatine kinase, muscle
1174	AP1S1	adaptor-related protein complex 1 sigma 1 subunit
1200	TPP1	tripeptidyl peptidase I

1213	CLTC	clathrin, heavy chain (Hc)
1306	COL15A1	collagen, type XV, alpha 1
1352	COX10	COX10 heme A:farnesyltransferase cytochrome c oxidase assembly factor
1355	COX15	cytochrome c oxidase assembly homolog 15 (yeast)
1398	CRK	v-crk avian sarcoma virus CT10 oncogene homolog
1465	CSRP1	cysteine and glycine rich protein 1
1523	CUX1	cut-like homeobox 1
1627	DBN1	drebrin 1
1727	CYB5R3	cytochrome b5 reductase 3
1730	DIAPH2	diaphanous related formin 2
1741	DLG3	discs, large homolog 3 (Drosophila)
1747	DLX3	distal-less homeobox 3
1757	SARDH	sarcosine dehydrogenase
1762	DMWD	dystrophia myotonica, WD repeat containing
1857	DVL3	dishevelled segment polarity protein 3
1861	TOR1A	torsin family 1, member A (torsin A)
1879	EBF1	early B-cell factor 1
1951	CELSR3	cadherin, EGF LAG seven-pass G-type receptor 3
1952	CELSR2	cadherin, EGF LAG seven-pass G-type receptor 2
1956	EGFR	epidermal growth factor receptor
1981	EIF4G1	eukaryotic translation initiation factor 4 gamma, 1
1995	ELAVL3	ELAV like neuron-specific RNA binding protein 3
2006	ELN	elastin
2046	EPHA8	EPH receptor A8
2077	ERF	Ets2 repressor factor
2137	EXTL3	exostosin-like glycosyltransferase 3
2185	PTK2B	protein tyrosine kinase 2 beta
2354	FOSB	FBJ murine osteosarcoma viral oncogene homolog B
2538	G6PC	glucose-6-phosphatase, catalytic subunit
2670	GFAP	glial fibrillary acidic protein
2676	GFRA3	GDNF family receptor alpha 3
2827	GPR3	G protein-coupled receptor 3
2840	GPR17	G protein-coupled receptor 17
2872	MKNK2	MAP kinase interacting serine/threonine kinase 2
2874	GPS2	G protein pathway suppressor 2
2878	GPX3	glutathione peroxidase 3
2879	GPX4	glutathione peroxidase 4
2890	GRIA1	glutamate receptor, ionotropic, AMPA 1
2903	GRIN2A	glutamate receptor, ionotropic, N-methyl D-aspartate 2A
2909	ARHGAP35	Rho GTPase activating protein 35
2997	GYS1	glycogen synthase 1 (muscle)
3005	H1F0	H1 histone family member 0

3149	HMGB3	high mobility group box 3
3159	HMGA1	high mobility group AT-hook 1
3170	FOXA2	forkhead box A2
3190	HNRNPK	heterogeneous nuclear ribonucleoprotein K
3213	HOXB3	homeobox B3
3223	HOXC6	homeobox C6
3229	HOXC13	homeobox C13
3339	HSPG2	heparan sulfate proteoglycan 2
3340	NDST1	N-deacetylase/N-sulfotransferase (heparan glucosaminyl) 1
3488	IGFBP5	insulin like growth factor binding protein 5
3549	IHH	indian hedgehog
3560	IL2RB	interleukin 2 receptor, beta
3692	EIF6	eukaryotic translation initiation factor 6
3707	ITPKB	inositol-trisphosphate 3-kinase B
3748	KCNC3	potassium channel, voltage gated Shaw related subfamily C, member 3
3756	KCNH1	potassium channel, voltage gated eag related subfamily H, member 1
3897	L1CAM	L1 cell adhesion molecule
4059	BCAM	basal cell adhesion molecule (Lutheran blood group)
4150	MAZ	MYC-associated zinc finger protein (purine-binding transcription factor)
4204	MECP2	methyl-CpG binding protein 2
4209	MEF2D	myocyte enhancer factor 2D
4215	MAP3K3	mitogen-activated protein kinase kinase kinase 3
4222	MEOX1	mesenchyme homeobox 1
4237	MFAP2	microfibrillar associated protein 2
4261	CIITA	class II, major histocompatibility complex, transactivator
4277	MICB	MHC class I polypeptide-related sequence B
4303	FOXO4	forkhead box O4
4324	MMP15	matrix metallopeptidase 15 (membrane-inserted)
4335	MNT	MAX network transcriptional repressor
4355	MPP2	membrane protein, palmitoylated 2
4478	MSN	moesin
4440	MSI1	musashi RNA binding protein 1
4647	MYO7A	myosin VIIA
4648	MYO7B	myosin VIIB
4656	MYOG	myogenin (myogenic factor 4)
4668	NAGA	N-acetylgalactosaminidase, alpha-
4763	NF1	neurofibromin 1
4782	NFIC	nuclear factor I/C (CCAAT-binding transcription factor)
4807	NHLH1	nescient helix-loop-helix 1
4851	NOTCH1	notch 1
4900	NRGN	neurogranin
4915	NTRK2	neurotrophic tyrosine kinase, receptor, type 2

4923	NTSR1	neurotensin receptor 1 (high affinity)
5029	P2RY2	purinergic receptor P2Y, G-protein coupled, 2
5045	FURIN	furin (paired basic amino acid cleaving enzyme)
5089	PBX2	pre-B-cell leukemia homeobox 2
5129	CDK18	cyclin-dependent kinase 18
5209	PFKFB3	6-phosphofructo-2-kinase/fructose-2,6-biphosphatase 3
5236	PGM1	phosphoglucomutase 1
5245	PHB	prohibitin
5326	PLAGL2	pleiomorphic adenoma gene-like 2
5360	PLTP	phospholipid transfer protein
5396	PRRX1	paired related homeobox 1
5478	PPIA	peptidylprolyl isomerase A (cyclophilin A)
5566	PRKACA	protein kinase, cAMP-dependent, alpha catalytic subunit
5582	PRKCG	protein kinase C, gamma
5589	PRKCSH	protein kinase C substrate 80K-H
5618	PRLR	prolactin receptor
5662	PSD	pleckstrin and Sec7 domain containing
5739	PTGIR	prostaglandin I2 (prostacyclin) receptor (IP)
5781	PTPN11	protein tyrosine phosphatase, non-receptor type 11
5784	PTPN14	protein tyrosine phosphatase, non-receptor type 14
5818	PVRL1	poliovirus receptor-related 1 (herpesvirus entry mediator C)
5869	RAB5B	RAB5B, member RAS oncogene family
5880	RAC2	ras-related C3 botulinum toxin substrate 2 (rho family, small GTP binding protein Rac2)
5947	RBP1	retinol binding protein 1, cellular
5957	RCVRN	recoverin
5977	DPF2	D4, zinc and double PHD fingers family 2
6196	RPS6KA2	ribosomal protein S6 kinase, 90kDa, polypeptide 2
6239	RREB1	ras responsive element binding protein 1
6272	SORT1	sortilin 1
6327	SCN2B	sodium channel, voltage gated, type II beta subunit
6334	SCN8A	sodium channel, voltage gated, type VIII alpha subunit
6338	SCNN1B	sodium channel, non voltage gated 1 beta subunit
6366	CCL21	chemokine (C-C motif) ligand 21
6376	CX3CL1	chemokine (C-X3-C motif) ligand 1
6439	SFTPB	surfactant protein B
6461	SHB	Src homology 2 domain containing adaptor protein B
6512	SLC1A7	solute carrier family 1 (glutamate transporter), member 7
6543	SLC8A2	solute carrier family 8 (sodium/calcium exchanger), member 2
6578	SLCO2A1	solute carrier organic anion transporter family member 2A1
6599	SMARCC1	SWI/SNF related, matrix associated, actin dependent regulator of chromatin, subfamily c, member 1
6601	SMARCC2	SWI/SNF related, matrix associated, actin dependent regulator of

		chromatin, subfamily c, member 2
6602	SMARCD1	SWI/SNF related, matrix associated, actin dependent regulator of chromatin, subfamily d, member 1
6612	SUMO3	small ubiquitin-like modifier 3
6649	SOD3	superoxide dismutase 3, extracellular
6663	SOX10	SRY-box 10
6666	SOX12	SRY-box 12
6768	ST14	suppression of tumorigenicity 14
6777	STAT5B	signal transducer and activator of transcription 5B
6781	STC1	stanniocalcin 1
6804	STX1A	syntaxin 1A
6844	VAMP2	vesicle associated membrane protein 2
6855	SYP	synaptophysin
6861	SYT5	synaptotagmin 5
6900	CNTN2	contactin 2 (axonal)
6911	TBX6	T-box 6
6943	TCF21	transcription factor 21
7009	TMBIM6	transmembrane BAX inhibitor motif containing 6
7023	TFAP4	transcription factor AP-4 (activating enhancer binding protein 4)
7030	TFE3	transcription factor binding to IGHM enhancer 3
7056	THBD	thrombomodulin
7090	TLE3	transducin like enhancer of split 3
7111	TMOD1	tropomodulin 1
7126	TNFAIP1	TNF alpha induced protein 1
7145	TNS1	tensin 1
7392	USF2	upstream transcription factor 2, c-fos interacting
7421	VDR	vitamin D (1,25- dihydroxyvitamin D3) receptor
7531	YWHAE	tyrosine 3-monooxygenase/tryptophan 5-monooxygenase activation protein, epsilon
7533	YWHAH	tyrosine 3-monooxygenase/tryptophan 5-monooxygenase activation protein, eta
7579	ZSCAN20	zinc finger and SCAN domain containing 20
7621	ZNF70	zinc finger protein 70
7745	ZKSCAN8	zinc finger with KRAB and SCAN domains 8
8000	PSCA	prostate stem cell antigen
8030	CCDC6	coiled-coil domain containing 6
8085	KMT2D	lysine (K)-specific methyltransferase 2D
8128	ST8SIA2	ST8 alpha-N-acetyl-neuraminide alpha-2,8-sialyltransferase 2
8153	RND2	Rho family GTPase 2
8227	AKAP17A	A-kinase anchoring protein 17A
8303	SNN	stannin
8325	FZD8	frizzled class receptor 8
8396	PIP4K2B	phosphatidylinositol-5-phosphate 4-kinase, type II, beta

8398	PLA2G6	phospholipase A2 group VI
8463	TEAD2	TEA domain family member 2
8514	KCNAB2	potassium channel, voltage gated subfamily A regulatory beta subunit 2
8525	DGKZ	diacylglycerol kinase, zeta
8554	PIAS1	protein inhibitor of activated STAT 1
8572	PDLIM4	PDZ and LIM domain 4
8633	UNC5C	unc-5 netrin receptor C
8659	ALDH4A1	aldehyde dehydrogenase 4 family member A1
8728	ADAM19	ADAM metallopeptidase domain 19
8927	BSN	bassoon presynaptic cytomatrix protein
8941	CDK5R2	cyclin-dependent kinase 5, regulatory subunit 2 (p39)
9028	RHBDL1	rhomboid, veinlet-like 1 (Drosophila)
9132	KCNQ4	potassium channel, voltage gated KQT-like subfamily Q, member 4
9138	ARHGEF1	Rho guanine nucleotide exchange factor 1
9253	NUMBL	numb homolog (Drosophila)-like
9254	CACNA2D2	calcium channel, voltage-dependent, alpha 2/delta subunit 2
9381	OTOF	otoferlin
9415	FADS2	fatty acid desaturase 2
9421	HAND1	heart and neural crest derivatives expressed 1
9453	GGPS1	geranylgeranyl diphosphate synthase 1
9468	PCYT1B	phosphate cytidylyltransferase 1, choline, beta
9472	AKAP6	A-kinase anchoring protein 6
9480	ONECUT2	one cut homeobox 2
9520	NPEPPS	aminopeptidase puromycin sensitive
9628	RGS6	regulator of G-protein signaling 6
9644	SH3PXD2A	SH3 and PX domains 2A
9651	PLCH2	phospholipase C eta 2
9658	ZNF516	zinc finger protein 516
9670	IPO13	importin 13
9761	MLEC	malectin
9776	ATG13	autophagy related 13
9777	TM9SF4	transmembrane 9 superfamily protein member 4
9905	SGSM2	small G protein signaling modulator 2
9938	ARHGAP25	Rho GTPase activating protein 25
9942	XYLB	xylulokinase homolog (H. influenzae)
9967	THRAP3	thyroid hormone receptor associated protein 3
10090	UST	uronyl-2-sulfotransferase
10096	ACTR3	ARP3 actin-related protein 3 homolog (yeast)
10174	SORBS3	sorbin and SH3 domain containing 3
10211	FLOT1	flotillin 1
10232	MSLN	mesothelin
10297	APC2	adenomatosis polyposis coli 2

10298	PAK4	p21 protein (Cdc42/Rac)-activated kinase 4
10311	DSCR3	Down syndrome critical region 3
10362	HMG20B	high mobility group 20B
10391	CORO2B	coronin, actin binding protein, 2B
10397	NDRG1	N-myc downstream regulated 1
10454	TAB1	TGF-beta activated kinase 1/MAP3K7 binding protein 1
10481	HOXB13	homeobox B13
10494	STK25	serine/threonine kinase 25
10498	CARM1	coactivator associated arginine methyltransferase 1
10525	HYOU1	hypoxia up-regulated 1
10562	OLFM4	olfactomedin 4
10645	CAMKK2	calcium/calmodulin-dependent protein kinase kinase 2, beta
10715	CERS1	ceramide synthase 1
10725	NFAT5	nuclear factor of activated T-cells 5, tonicity-responsive
10743	RAI1	retinoic acid induced 1
10766	TOB2	transducer of ERBB2, 2
10771	ZMYND11	zinc finger, MYND-type containing 11
10788	IQGAP2	IQ motif containing GTPase activating protein 2
10814	CPLX2	complexin 2
10815	CPLX1	complexin 1
10867	TSPAN9	tetraspanin 9
10893	MMP24	matrix metallopeptidase 24 (membrane-inserted)
10945	KDELR1	KDEL (Lys-Asp-Glu-Leu) endoplasmic reticulum protein retention receptor 1
11135	CDC42EP1	CDC42 effector protein (Rho GTPase binding) 1
11155	LDB3	LIM domain binding 3
11170	FAM107A	family with sequence similarity 107 member A
11176	BAZ2A	bromodomain adjacent to zinc finger domain 2A
11240	PADI2	peptidyl arginine deiminase, type II
11248	NXPH3	neurexophilin 3
11261	CHP1	calcineurin-like EF-hand protein 1
11273	ATXN2L	ataxin 2-like
22801	ITGA11	integrin alpha 11
22826	DNAJC8	DnaJ heat shock protein family (Hsp40) member C8
22888	UBOX5	U-box domain containing 5
22931	RAB18	RAB18, member RAS oncogene family
22936	ELL2	elongation factor, RNA polymerase II, 2
22953	P2RX2	purinergic receptor P2X, ligand gated ion channel, 2
22992	KDM2A	lysine (K)-specific demethylase 2A
22993	HMGXB3	HMG box domain containing 3
23034	SAMD4A	sterile alpha motif domain containing 4A
23038	WDTC1	WD and tetratricopeptide repeats 1
23046	KIF21B	kinesin family member 21B

23090	ZNF423	zinc finger protein 423
23096	IQSEC2	IQ motif and Sec7 domain 2
23109	DDN	dendrin
23119	HIC2	hypermethylated in cancer 2
23126	POGZ	pogo transposable element with ZNF domain
23211	ZC3H4	zinc finger CCCH-type containing 4
23217	ZFR2	zinc finger RNA binding protein 2
23247	KIAA0556	KIAA0556
23251	KIAA1024	KIAA1024
23264	ZC3H7B	zinc finger CCCH-type containing 7B
23270	TSPYL4	TSPY-like 4
23276	KLHL18	kelch like family member 18
23293	SMG6	SMG6 nonsense mediated mRNA decay factor
23322	RPGRIP1L	RPGRIP1-like
23334	SZT2	seizure threshold 2 homolog (mouse)
23373	CRTC1	CREB regulated transcription coactivator 1
23380	SRGAP2	SLIT-ROBO Rho GTPase activating protein 2
23403	FBXO46	F-box protein 46
23466	CBX6	chromobox 6
23468	CBX5	chromobox 5
23476	BRD4	bromodomain containing 4
23500	DAAM2	dishevelled associated activator of morphogenesis 2
23598	PATZ1	POZ (BTB) and AT hook containing zinc finger 1
25758	KIAA1549L	KIAA1549-like
25789	TMEM59L	transmembrane protein 59 like
25840	METTL7A	methyltransferase like 7A
25946	ZNF385A	zinc finger protein 385A
25970	SH2B1	SH2B adaptor protein 1
26000	TBC1D10B	TBC1 domain family member 10B
26011	TENM4	teneurin transmembrane protein 4
26034	IPCEF1	interaction protein for cytohesin exchange factors 1
26051	PPP1R16B	protein phosphatase 1 regulatory subunit 16B
26099	SZRD1	SUZ RNA binding domain containing 1
26145	IRF2BP1	interferon regulatory factor 2 binding protein 1
26468	LHX6	LIM homeobox 6
26469	PTPN18	protein tyrosine phosphatase, non-receptor type 18 (brain-derived)
26507	CNNM1	cyclin and CBS domain divalent metal cation transport mediator 1
26523	AGO1	argonaute RISC catalytic component 1
27112	FAM155B	family with sequence similarity 155 member B
27128	CYTH4	cytohesin 4
27315	PGAP2	post-GPI attachment to proteins 2
27351	DESI1	desumoylating isopeptidase 1
27433	TOR2A	torsin family 2, member A

27434	POLM	polymerase (DNA directed), mu
28511	NKIRAS2	NFKB inhibitor interacting Ras-like 2
28964	GIT1	G protein-coupled receptor kinase interacting ArfGAP 1
28974	C19orf53	chromosome 19 open reading frame 53
29115	SAP30BP	SAP30 binding protein
29780	PARVB	parvin beta
29924	EPN1	epsin 1
50488	MINK1	misshapen-like kinase 1
51043	ZBTB7B	zinc finger and BTB domain containing 7B
51082	POLR1D	polymerase (RNA) I polypeptide D
51108	METTL9	methyltransferase like 9
51341	ZBTB7A	zinc finger and BTB domain containing 7A
51441	YTHDF2	YTH N(6)-methyladenosine RNA binding protein 2
51564	HDAC7	histone deacetylase 7
51592	TRIM33	tripartite motif containing 33
51629	SLC25A39	solute carrier family 25, member 39
51725	FBXO40	F-box protein 40
51752	ERAP1	endoplasmic reticulum aminopeptidase 1
53405	CLIC5	chloride intracellular channel 5
53834	FGFRL1	fibroblast growth factor receptor-like 1
53944	CSNK1G1	casein kinase 1, gamma 1
54331	GNG2	guanine nucleotide binding protein (G protein), gamma 2
54509	RHOF	ras homolog family member F (in filopodia)
54741	LEPROT	leptin receptor overlapping transcript
54788	DNAJB12	DnaJ heat shock protein family (Hsp40) member B12
54868	TMEM104	transmembrane protein 104
54957	TXNL4B	thioredoxin-like 4B
54976	C20orf27	chromosome 20 open reading frame 27
55036	CCDC40	coiled-coil domain containing 40
55065	SLC52A1	solute carrier family 52 (riboflavin transporter), member 1
55095	SAMD4B	sterile alpha motif domain containing 4B
55176	SEC61A2	Sec61 translocon alpha 2 subunit
55224	ETNK2	ethanolamine kinase 2
55243	KIRREL	kin of IRRE like (Drosophila)
55295	KLHL26	kelch like family member 26
55322	C5orf22	chromosome 5 open reading frame 22
55362	TMEM63B	transmembrane protein 63B
55577	NAGK	N-acetylglucosamine kinase
55603	FAM46A	family with sequence similarity 46 member A
55604	LRRC16A	leucine rich repeat containing 16A
55640	FLVCR2	feline leukemia virus subgroup C cellular receptor family member 2
55652	SLC48A1	solute carrier family 48 (heme transporter), member 1
55690	PACS1	phosphofurin acidic cluster sorting protein 1

55793	FAM63A	family with sequence similarity 63 member A
55810	FOXJ2	forkhead box J2
55920	RCC2	regulator of chromosome condensation 2
56000	NXF3	nuclear RNA export factor 3
56413	LTB4R2	leukotriene B4 receptor 2
56834	GPR137	G protein-coupled receptor 137
56852	RAD18	RAD18 E3 ubiquitin protein ligase
56965	PARP6	poly(ADP-ribose) polymerase family member 6
56970	ATXN7L3	ataxin 7-like 3
57026	PDXP	pyridoxal (pyridoxine, vitamin B6) phosphatase
57111	RAB25	RAB25, member RAS oncogene family
57154	SMURF1	SMAD specific E3 ubiquitin protein ligase 1
57217	TTC7A	tetratricopeptide repeat domain 7A
57449	PLEKHG5	pleckstrin homology and RhoGEF domain containing G5
57452	GALNT16	polypeptide N-acetylgalactosaminyltransferase 16
57453	DSCAML1	Down syndrome cell adhesion molecule like 1
57458	TMCC3	transmembrane and coiled-coil domain family 3
57473	ZNF512B	zinc finger protein 512B
57605	PITPNM2	phosphatidylinositol transfer protein, membrane associated 2
57608	KIAA1462	KIAA1462
57611	ISLR2	immunoglobulin superfamily containing leucine-rich repeat 2
57715	SEMA4G	sema domain, immunoglobulin domain (Ig), transmembrane domain (TM) and short cytoplasmic domain, (semaphorin) 4G
57834	CYP4F11	cytochrome P450, family 4, subfamily F, polypeptide 11
57863	CADM3	cell adhesion molecule 3
58526	MID1IP1	MID1 interacting protein 1
59269	HIVEP3	human immunodeficiency virus type I enhancer binding protein 3
59274	MESDC1	mesoderm development candidate 1
60677	CELF6	CUGBP, Elav-like family member 6
63910	SLC17A9	solute carrier family 17 (vesicular nucleotide transporter), member 9
63974	NEUROD6	neuronal differentiation 6
64067	NPAS3	neuronal PAS domain protein 3
64129	TINAGL1	tubulointerstitial nephritis antigen-like 1
64137	ABCG4	ATP binding cassette subfamily G member 4
64599	GIGYF1	GRB10 interacting GYF protein 1
64689	GORASP1	golgi reassembly stacking protein 1
64834	ELOVL1	ELOVL fatty acid elongase 1
65078	RTN4R	reticulon 4 receptor
79016	DDA1	DET1 and DDB1 associated 1
79033	ERI3	ERI1 exoribonuclease family member 3
79065	ATG9A	autophagy related 9A
79152	FA2H	fatty acid 2-hydroxylase
79178	THTPA	thiamine triphosphatase

79365	BHLHE41	basic helix-loop-helix family member e41
79712	GTDC1	glycosyltransferase like domain containing 1
79751	SLC25A22	solute carrier family 25 (mitochondrial carrier: glutamate), member 22
79789	CLMN	calmin (calponin-like, transmembrane)
79837	PIP4K2C	phosphatidylinositol-5-phosphate 4-kinase, type II, gamma
79875	THSD4	thrombospondin type 1 domain containing 4
79991	OBFC1	oligonucleotide/oligosaccharide-binding fold containing 1
80162	ATHL1	ATH1, acid trehalase-like 1 (yeast)
80320	SP6	Sp6 transcription factor
80725	SRCIN1	SRC kinase signaling inhibitor 1
80774	LIMD2	LIM domain containing 2
80790	CMIP	c-Maf inducing protein
81619	TSPAN14	tetraspanin 14
81848	SPRY4	sprouty RTK signaling antagonist 4
83394	PITPNM3	PITPNM family member 3
83637	ZMIZ2	zinc finger, MIZ-type containing 2
83714	NRIP2	nuclear receptor interacting protein 2
83716	CRISPLD2	cysteine-rich secretory protein LCCL domain containing 2
84329	HVCN1	hydrogen voltage gated channel 1
84447	SYVN1	synoviolin 1
84530	SRRM4	serine/arginine repetitive matrix 4
84630	TTBK1	tau tubulin kinase 1
84649	DGAT2	diacylglycerol O-acyltransferase 2
84687	PPP1R9B	protein phosphatase 1 regulatory subunit 9B
84726	PRRC2B	proline-rich coiled-coil 2B
84929	FIBCD1	fibrinogen C domain containing 1
85352	KIAA1644	KIAA1644
85414	SLC45A3	solute carrier family 45, member 3
85446	ZFHX2	zinc finger homeobox 2
89790	SIGLEC10	sialic acid binding Ig-like lectin 10
89797	NAV2	neuron navigator 2
89848	FCHSD1	FCH and double SH3 domains 1
89853	MVB12B	multivesicular body subunit 12B
90121	TSR2	TSR2, 20S rRNA accumulation, homolog (S. cerevisiae)
90139	TSPAN18	tetraspanin 18
90362	FAM110B	family with sequence similarity 110 member B
90407	TMEM41A	transmembrane protein 41A
90933	TRIM41	tripartite motif containing 41
90993	CREB3L1	cAMP responsive element binding protein 3-like 1
91608	RASL10B	RAS-like, family 10, member B
91748	ELMSAN1	ELM2 and Myb/SANT-like domain containing 1
92154	MTSS1L	metastasis suppressor 1-like
92558	CCDC64	coiled-coil domain containing 64

92736	OTOP2	otopetrin 2
92949	ADAMTSL1	ADAMTS like 1
93145	OLFM2	olfactomedin 2
93380	MMGT1	membrane magnesium transporter 1
93517	SDR42E1	short chain dehydrogenase/reductase family 42E, member 1
112939	NACC1	nucleus accumbens associated 1, BEN and BTB (POZ) domain containing
113189	CHST14	carbohydrate (N-acetylgalactosamine 4-0) sulfotransferase 14
113829	SLC35A4	solute carrier family 35, member A4
114787	GPRIN1	G protein regulated inducer of neurite outgrowth 1
114881	OSBPL7	oxysterol binding protein like 7
114897	C1QTNF1	C1q and tumor necrosis factor related protein 1
115572	FAM46B	family with sequence similarity 46 member B
116113	FOXP4	forkhead box P4
116983	ACAP3	ArfGAP with coiled-coil, ankyrin repeat and PH domains 3
117581	TWIST2	twist family bHLH transcription factor 2
121512	FGD4	FYVE, RhoGEF and PH domain containing 4
124460	SNX20	sorting nexin 20
124583	CANT1	calcium activated nucleotidase 1
124590	USH1G	Usher syndrome 1G (autosomal recessive)
124857	WFIKKN2	WAP, follistatin/kazal, immunoglobulin, kunitz and netrin domain containing 2
124930	ANKRD13B	ankyrin repeat domain 13B
124935	SLC43A2	solute carrier family 43 (amino acid system L transporter), member 2
124976	SPNS2	spinster homolog 2 (Drosophila)
125058	TBC1D16	TBC1 domain family, member 16
125950	RAVER1	ribonucleoprotein, PTB-binding 1
126014	OSCAR	osteoclast associated, immunoglobulin-like receptor
126393	HSPB6	heat shock protein family B (small) member 6
126410	CYP4F22	cytochrome P450, family 4, subfamily F, polypeptide 22
126549	ANKLE1	ankyrin repeat and LEM domain containing 1
128434	VSTM2L	V-set and transmembrane domain containing 2 like
132228	LSMEM2	leucine-rich single-pass membrane protein 2
134266	GRPEL2	GrpE-like 2, mitochondrial (E. coli)
134288	TMEM174	transmembrane protein 174
135656	DPCR1	diffuse panbronchiolitis critical region 1
138162	C9orf116	chromosome 9 open reading frame 116
140885	SIRPA	signal-regulatory protein alpha
145270	PRIMA1	proline rich membrane anchor 1
145567	TTC7B	tetratricopeptide repeat domain 7B
146691	TOM1L2	target of myb1 like 2 membrane trafficking protein
147339	C18orf25	chromosome 18 open reading frame 25
148789	B3GALNT2	beta-1,3-N-acetylgalactosaminyltransferase 2

150372	NFAM1	NFAT activating protein with ITAM motif 1
158471	PRUNE2	prune homolog 2 (Drosophila)
162466	PHOSPHO1	phosphatase, orphan 1
162494	RHBDL3	rhomboid, veinlet-like 3 (Drosophila)
162514	TRPV3	transient receptor potential cation channel, subfamily V, member 3
164633	CABP7	calcium binding protein 7
167826	OLIG3	oligodendrocyte transcription factor 3
170575	GIMAP1	GTPase, IMAP family member 1
171017	ZNF384	zinc finger protein 384
196527	ANO6	anoctamin 6
199870	FAM76A	family with sequence similarity 76 member A
199964	TMEM61	transmembrane protein 61
201514	ZNF584	zinc finger protein 584
203259	FAM219A	family with sequence similarity 219 member A
219654	ZCCHC24	zinc finger, CCHC domain containing 24
219699	UNC5B	unc-5 netrin receptor B
219855	SLC37A2	solute carrier family 37 (glucose-6-phosphate transporter), member 2
219995	MS4A15	membrane-spanning 4-domains, subfamily A, member 15
220074	LRTOMT	leucine rich transmembrane and O-methyltransferase domain containing
221687	RNF182	ring finger protein 182
221935	SDK1	sidekick cell adhesion molecule 1
222865	TMEM130	transmembrane protein 130
246330	PELI3	pellino E3 ubiquitin protein ligase family member 3
253461	ZBTB38	zinc finger and BTB domain containing 38
254263	CNIH2	cornichon family AMPA receptor auxiliary protein 2
254428	SLC41A1	solute carrier family 41 (magnesium transporter), member 1
266727	MDGA1	MAM domain containing glycosylphosphatidylinositol anchor 1
266743	NPAS4	neuronal PAS domain protein 4
283337	ZNF740	zinc finger protein 740
283455	KSR2	kinase suppressor of ras 2
283638	CEP170B	centrosomal protein 170B
284106	CISD3	CDGSH iron sulfur domain 3
284340	CXCL17	chemokine (C-X-C motif) ligand 17
284613	CYB561D1	cytochrome b561 family member D1
285590	SH3PXD2B	SH3 and PX domains 2B
286336	FAM78A	family with sequence similarity 78 member A
338324	S100A7A	S100 calcium binding protein A7A
338917	VSX2	visual system homeobox 2
339122	RAB43	RAB43, member RAS oncogene family
340061	TMEM173	transmembrane protein 173
342933	ZSCAN5B	zinc finger and SCAN domain containing 5B
347365	ITIH6	inter-alpha-trypsin inhibitor heavy chain family member 6

374655	ZNF710	zinc finger protein 710
375057	C1orf95	chromosome 1 open reading frame 95
375790	AGRN	agrin
387509	GPR153	G protein-coupled receptor 153
388115	C15orf52	chromosome 15 open reading frame 52
388336	SHISA6	shisa family member 6
388969	C2orf68	chromosome 2 open reading frame 68
399687	MYO18A	myosin XVIIIA
399947	C11orf87	chromosome 11 open reading frame 87
399979	SNX19	sorting nexin 19
402665	IGLON5	IgLON family member 5
404037	HAPLN4	hyaluronan and proteoglycan link protein 4
441631	TSPAN11	tetraspanin 11
642938	FAM196A	family with sequence similarity 196 member A
643866	CBLN3	cerebellin 3 precursor
643988	C1orf233	chromosome 1 open reading frame 233
644150	WIPF3	WAS/WASL interacting protein family member 3
653509	SFTPA1	surfactant protein A1
654790	PCP4L1	Purkinje cell protein 4 like 1
9762	LZTS3	leucine zipper, putative tumor suppressor family member 3
23630	KCNE5	potassium channel, voltage gated subfamily E regulatory beta subunit 5
123591	TMEM266	transmembrane protein 266

Supplementary table 4: A list of KEGG pathways of predicted and validated target genes of hsa-miR-608

Term	Count	PValue
hsa04810:Regulation of actin cytoskeleton	18	4.96E-04
hsa04722:Neurotrophin signaling pathway	13	5.87E-04
hsa05220:Chronic myeloid leukemia	10	6.12E-04
hsa05200:Pathways in cancer	23	7.44E-04
hsa04144:Endocytosis	16	7.81E-04
hsa04360:Axon guidance	13	8.38E-04
hsa05210:Colorectal cancer	10	1.40E-03
hsa04010:MAPK signaling pathway	19	2.18E-03
hsa04510:Focal adhesion	15	5.07E-03
hsa04720:Long-term potentiation	8	5.92E-03
hsa04012:ErbB signaling pathway	9	6.62E-03
hsa05212:Pancreatic cancer	8	8.08E-03
hsa04370:VEGF signaling pathway	8	1.00E-02
hsa04020:Calcium signaling pathway	13	1.09E-02
hsa04666:Fc gamma R-mediated phagocytosis	9	1.11E-02
hsa05214:Glioma	7	1.53E-02

hsa00564:Glycerophospholipid metabolism	7	2.16E-02
hsa04310:Wnt signaling pathway	11	2.33E-02
hsa05211:Renal cell carcinoma	7	2.46E-02
hsa04630:Jak-STAT signaling pathway	11	2.73E-02
hsa05110:Vibrio cholerae infection	6	3.34E-02
hsa04670:Leukocyte transendothelial migration	9	3.59E-02
hsa04520:Adherens junction	7	3.70E-02
hsa04912:GnRH signaling pathway	8	3.80E-02
hsa04062:Chemokine signaling pathway	12	3.82E-02

Supplementary table 5: A list of Gene Ontology (GO) of predicted and validated target genes of hsa-miR-608

Category	Term	Count	PValue
GOTERM_BP_FAT	GO:0009891~positive regulation of biosynthetic process	51	1.26E-07
GOTERM_BP_FAT	GO:0010557~positive regulation of macromolecule biosynthetic process	49	1.27E-07
GOTERM_BP_FAT	GO:0010628~positive regulation of gene expression	45	1.85E-07
GOTERM_BP_FAT	GO:0031328~positive regulation of cellular biosynthetic process	50	2.02E-07
GOTERM_BP_FAT	GO:0045941~positive regulation of transcription	44	2.13E-07
GOTERM_BP_FAT	GO:0051173~positive regulation of nitrogen compound metabolic process	47	5.15E-07
GOTERM_BP_FAT	GO:0045893~positive regulation of transcription, DNA-dependent	38	1.04E-06
GOTERM_BP_FAT	GO:0051254~positive regulation of RNA metabolic process	38	1.29E-06
GOTERM_BP_FAT	GO:0045935~positive regulation of nucleobase, nucleoside, nucleotide and nucleic acid metabolic process	45	1.31E-06
GOTERM_BP_FAT	GO:0006357~regulation of transcription from RNA polymerase II promoter	49	2.69E-06
GOTERM_BP_FAT	GO:0030182~neuron differentiation	35	2.81E-06
GOTERM_BP_FAT	GO:0030030~cell projection organization	31	4.16E-06
GOTERM_BP_FAT	GO:0010604~positive regulation of macromolecule metabolic process	54	5.50E-06
GOTERM_BP_FAT	GO:0048666~neuron development	28	1.93E-05
GOTERM_BP_FAT	GO:0045449~regulation of transcription	121	2.69E-05
GOTERM_BP_FAT	GO:0045944~positive regulation of transcription from RNA polymerase II promoter	29	3.59E-05
GOTERM_BP_FAT	GO:0051252~regulation of RNA metabolic process	90	4.49E-05

GOTERM_BP_FAT	GO:0006355~regulation of transcription, DNA-dependent	88	5.73E-05
GOTERM_BP_FAT	GO:0031175~neuron projection development	21	2.96E-04
GOTERM_BP_FAT	GO:0048598~embryonic morphogenesis	23	5.00E-04
GOTERM_BP_FAT	GO:0007423~sensory organ development	19	5.46E-04
GOTERM_BP_FAT	GO:0007242~intracellular signaling cascade	63	6.67E-04
GOTERM_BP_FAT	GO:0032990~cell part morphogenesis	20	7.81E-04
GOTERM_BP_FAT	GO:0006350~transcription	94	1.13E-03
GOTERM_BP_FAT	GO:0007169~transmembrane receptor protein tyrosine kinase signaling pathway	18	1.14E-03
GOTERM_BP_FAT	GO:0050773~regulation of dendrite development	5	1.16E-03
GOTERM_BP_FAT	GO:0043009~chordate embryonic development	23	1.34E-03
GOTERM_BP_FAT	GO:0048667~cell morphogenesis involved in neuron differentiation	17	1.44E-03
GOTERM_BP_FAT	GO:0009792~embryonic development ending in birth or egg hatching	23	1.50E-03
GOTERM_BP_FAT	GO:0046578~regulation of Ras protein signal transduction	17	1.53E-03
GOTERM_BP_FAT	GO:0001944~vasculature development	19	1.57E-03
GOTERM_BP_FAT	GO:0001889~liver development	8	1.61E-03
GOTERM_BP_FAT	GO:0030856~regulation of epithelial cell differentiation	6	2.28E-03
GOTERM_BP_FAT	GO:0042490~mechanoreceptor differentiation	6	2.28E-03
GOTERM_BP_FAT	GO:0060541~respiratory system development	11	2.84E-03
GOTERM_BP_FAT	GO:0001775~cell activation	20	2.92E-03
GOTERM_BP_FAT	GO:0032989~cellular component morphogenesis	25	2.92E-03
GOTERM_BP_FAT	GO:0001568~blood vessel development	18	2.95E-03
GOTERM_BP_FAT	GO:0048858~cell projection morphogenesis	18	2.95E-03
GOTERM_BP_FAT	GO:0070141~response to UV-A	3	3.14E-03
GOTERM_BP_FAT	GO:0010551~regulation of specific transcription from RNA polymerase II promoter	10	3.63E-03
GOTERM_BP_FAT	GO:0002521~leukocyte differentiation	12	3.83E-03
GOTERM_BP_FAT	GO:0051056~regulation of small GTPase mediated signal transduction	18	3.93E-03
GOTERM_BP_FAT	GO:0048568~embryonic organ development	14	4.35E-03
GOTERM_BP_FAT	GO:0048546~digestive tract morphogenesis	5	4.36E-03
GOTERM_BP_FAT	GO:0055123~digestive system development	5	4.36E-03
GOTERM_BP_FAT	GO:0048812~neuron projection morphogenesis	16	4.43E-03
GOTERM_BP_FAT	GO:0007167~enzyme linked receptor protein signaling pathway	22	4.44E-03
GOTERM_BP_FAT	GO:0001503~ossification	11	4.46E-03
GOTERM_BP_FAT	GO:0032583~regulation of gene-specific transcription	12	4.55E-03

GOTERM_BP_FAT	GO:0031668~cellular response to extracellular stimulus	8	4.78E-03
GOTERM_BP_FAT	GO:0030324~lung development	10	5.12E-03
GOTERM_BP_FAT	GO:0007243~protein kinase cascade	23	5.22E-03
GOTERM_BP_FAT	GO:0021700~developmental maturation	10	5.83E-03
GOTERM_BP_FAT	GO:0035295~tube development	16	5.95E-03
GOTERM_BP_FAT	GO:0045321~leukocyte activation	17	6.13E-03
GOTERM_BP_FAT	GO:0030323~respiratory tube development	10	6.22E-03
GOTERM_BP_FAT	GO:0043066~negative regulation of apoptosis	22	6.32E-03
GOTERM_BP_FAT	GO:0046777~protein amino acid autophosphorylation	9	6.61E-03
GOTERM_BP_FAT	GO:0030098~lymphocyte differentiation	10	6.62E-03
GOTERM_BP_FAT	GO:0000904~cell morphogenesis involved in differentiation	17	6.64E-03
GOTERM_BP_FAT	GO:0009895~negative regulation of catabolic process	6	6.76E-03
GOTERM_BP_FAT	GO:0000902~cell morphogenesis	22	6.85E-03
GOTERM_BP_FAT	GO:0060348~bone development	11	7.14E-03
GOTERM_BP_FAT	GO:0060113~inner ear receptor cell differentiation	5	7.16E-03
GOTERM_BP_FAT	GO:0048592~eye morphogenesis	8	7.23E-03
GOTERM_BP_FAT	GO:0043069~negative regulation of programmed cell death	22	7.47E-03
GOTERM_BP_FAT	GO:0000165~MAPKKK cascade	14	7.59E-03
GOTERM_BP_FAT	GO:0060548~negative regulation of cell death	22	7.76E-03
GOTERM_BP_FAT	GO:0010629~negative regulation of gene expression	28	8.35E-03
GOTERM_BP_FAT	GO:0031344~regulation of cell projection organization	9	8.66E-03
GOTERM_BP_FAT	GO:0016481~negative regulation of transcription	26	9.04E-03
GOTERM_BP_FAT	GO:0016477~cell migration	18	9.52E-03
GOTERM_BP_FAT	GO:0051174~regulation of phosphorus metabolic process	27	9.72E-03
GOTERM_BP_FAT	GO:0019220~regulation of phosphate metabolic process	27	9.72E-03
GOTERM_BP_FAT	GO:0051094~positive regulation of developmental process	18	1.02E-02
GOTERM_BP_FAT	GO:0031327~negative regulation of cellular biosynthetic process	30	1.02E-02
GOTERM_BP_FAT	GO:0007409~axonogenesis	14	1.11E-02
GOTERM_BP_FAT	GO:0001654~eye development	11	1.15E-02
GOTERM_BP_FAT	GO:0001649~osteoblast differentiation	6	1.16E-02
GOTERM_BP_FAT	GO:0045596~negative regulation of cell	15	1.18E-02

	differentiation		
GOTERM_BP_FAT	GO:0048562~embryonic organ morphogenesis	11	1.21E-02
GOTERM_BP_FAT	GO:0019058~viral infectious cycle	6	1.27E-02
GOTERM_BP_FAT	GO:0048706~embryonic skeletal system development	8	1.29E-02
GOTERM_BP_FAT	GO:0010558~negative regulation of macromolecule biosynthetic process	29	1.32E-02
GOTERM_BP_FAT	GO:0030029~actin filament-based process	16	1.32E-02
GOTERM_BP_FAT	GO:0006928~cell motion	26	1.34E-02
GOTERM_BP_FAT	GO:0001890~placenta development	7	1.34E-02
GOTERM_BP_FAT	GO:0009890~negative regulation of biosynthetic process	30	1.35E-02
GOTERM_BP_FAT	GO:0045892~negative regulation of transcription, DNA-dependent	21	1.35E-02
GOTERM_BP_FAT	GO:0009991~response to extracellular stimulus	15	1.36E-02
GOTERM_BP_FAT	GO:0043549~regulation of kinase activity	21	1.39E-02
GOTERM_BP_FAT	GO:0050804~regulation of synaptic transmission	11	1.39E-02
GOTERM_BP_FAT	GO:0046649~lymphocyte activation	14	1.40E-02
GOTERM_BP_FAT	GO:0042981~regulation of apoptosis	39	1.46E-02
GOTERM_BP_FAT	GO:0051253~negative regulation of RNA metabolic process	21	1.60E-02
GOTERM_BP_FAT	GO:0010605~negative regulation of macromolecule metabolic process	36	1.62E-02
GOTERM_BP_FAT	GO:0043067~regulation of programmed cell death	39	1.67E-02
GOTERM_BP_FAT	GO:0010243~response to organic nitrogen	7	1.68E-02
GOTERM_BP_FAT	GO:0030036~actin cytoskeleton organization	15	1.68E-02
GOTERM_BP_FAT	GO:0050850~positive regulation of calcium-mediated signaling	4	1.69E-02
GOTERM_BP_FAT	GO:0048547~gut morphogenesis	4	1.69E-02
GOTERM_BP_FAT	GO:0042177~negative regulation of protein catabolic process	4	1.69E-02
GOTERM_BP_FAT	GO:0010941~regulation of cell death	39	1.76E-02
GOTERM_BP_FAT	GO:0045597~positive regulation of cell differentiation	15	1.87E-02
GOTERM_BP_FAT	GO:0045859~regulation of protein kinase activity	20	1.91E-02
GOTERM_BP_FAT	GO:0042325~regulation of phosphorylation	25	1.93E-02
GOTERM_BP_FAT	GO:0030217~T cell differentiation	7	1.93E-02
GOTERM_BP_FAT	GO:0048754~branching morphogenesis of a tube	7	1.93E-02
GOTERM_BP_FAT	GO:0042491~auditory receptor cell differentiation	4	1.97E-02
GOTERM_BP_FAT	GO:0060117~auditory receptor cell development	3	2.01E-02

GOTERM_BP_FAT	GO:0051172~negative regulation of nitrogen compound metabolic process	27	2.06E-02
GOTERM_BP_FAT	GO:0051338~regulation of transferase activity	21	2.07E-02
GOTERM_BP_FAT	GO:0048514~blood vessel morphogenesis	14	2.17E-02
GOTERM_BP_FAT	GO:0051969~regulation of transmission of nerve impulse	11	2.28E-02
GOTERM_BP_FAT	GO:0045058~T cell selection	4	2.29E-02
GOTERM_BP_FAT	GO:0051325~interphase	9	2.30E-02
GOTERM_BP_FAT	GO:0007264~small GTPase mediated signal transduction	18	2.32E-02
GOTERM_BP_FAT	GO:0030097~hemopoiesis	15	2.33E-02
GOTERM_BP_FAT	GO:0035239~tube morphogenesis	10	2.37E-02
GOTERM_BP_FAT	GO:0051960~regulation of nervous system development	13	2.43E-02
GOTERM_BP_FAT	GO:0048870~cell motility	18	2.46E-02
GOTERM_BP_FAT	GO:0051674~localization of cell	18	2.46E-02
GOTERM_BP_FAT	GO:0050848~regulation of calcium-mediated signaling	4	2.63E-02
GOTERM_BP_FAT	GO:0045737~positive regulation of cyclin-dependent protein kinase activity	3	2.63E-02
GOTERM_BP_FAT	GO:0031670~cellular response to nutrient	3	2.63E-02
GOTERM_BP_FAT	GO:0031016~pancreas development	5	2.67E-02
GOTERM_BP_FAT	GO:0010975~regulation of neuron projection development	7	2.68E-02
GOTERM_BP_FAT	GO:0031644~regulation of neurological system process	11	2.90E-02
GOTERM_BP_FAT	GO:0007585~respiratory gaseous exchange	5	2.92E-02
GOTERM_BP_FAT	GO:0001776~leukocyte homeostasis	5	2.92E-02
GOTERM_BP_FAT	GO:0045884~regulation of survival gene product expression	4	2.99E-02
GOTERM_BP_FAT	GO:0001701~in utero embryonic development	12	3.02E-02
GOTERM_BP_FAT	GO:0045934~negative regulation of nucleobase, nucleoside, nucleotide and nucleic acid metabolic process	26	3.05E-02
GOTERM_BP_FAT	GO:0045664~regulation of neuron differentiation	10	3.07E-02
GOTERM_BP_FAT	GO:0000079~regulation of cyclin-dependent protein kinase activity	6	3.13E-02
GOTERM_BP_FAT	GO:0009314~response to radiation	13	3.20E-02
GOTERM_BP_FAT	GO:0042135~neurotransmitter catabolic process	3	3.31E-02
GOTERM_BP_FAT	GO:0060119~inner ear receptor cell development	3	3.31E-02
GOTERM_BP_FAT	GO:0048732~gland development	10	3.34E-02
GOTERM_BP_FAT	GO:0009628~response to abiotic stimulus	20	3.38E-02
GOTERM_BP_FAT	GO:0019059~initiation of viral infection	4	3.38E-02

GOTERM_BP_FAT	GO:0001763~morphogenesis of a branching structure	7	3.40E-02
GOTERM_BP_FAT	GO:0045767~regulation of anti-apoptosis	5	3.48E-02
GOTERM_BP_FAT	GO:0000082~G1/S transition of mitotic cell cycle	6	3.59E-02
GOTERM_BP_FAT	GO:0009416~response to light stimulus	10	3.76E-02
GOTERM_BP_FAT	GO:0048567~ectodermal gut morphogenesis	3	4.05E-02
GOTERM_BP_FAT	GO:0048708~astrocyte differentiation	3	4.05E-02
GOTERM_BP_FAT	GO:0007439~ectodermal gut development	3	4.05E-02
GOTERM_BP_FAT	GO:0046486~glycerolipid metabolic process	11	4.05E-02
GOTERM_BP_FAT	GO:0033674~positive regulation of kinase activity	14	4.06E-02
GOTERM_BP_FAT	GO:0006892~post-Golgi vesicle-mediated transport	6	4.08E-02
GOTERM_BP_FAT	GO:0010647~positive regulation of cell communication	18	4.31E-02
GOTERM_BP_FAT	GO:0010553~negative regulation of specific transcription from RNA polymerase II promoter	5	4.43E-02
GOTERM_BP_FAT	GO:0048839~inner ear development	7	4.47E-02
GOTERM_BP_FAT	GO:0050767~regulation of neurogenesis	11	4.65E-02
GOTERM_BP_FAT	GO:0002694~regulation of leukocyte activation	11	4.65E-02
GOTERM_BP_FAT	GO:0048534~hemopoietic or lymphoid organ development	15	4.73E-02
GOTERM_BP_FAT	GO:0022404~molting cycle process	5	4.77E-02
GOTERM_BP_FAT	GO:0001942~hair follicle development	5	4.77E-02
GOTERM_BP_FAT	GO:0048663~neuron fate commitment	5	4.77E-02
GOTERM_BP_FAT	GO:0022405~hair cycle process	5	4.77E-02
GOTERM_BP_FAT	GO:0046902~regulation of mitochondrial membrane permeability	3	4.84E-02
GOTERM_BP_FAT	GO:0043586~tongue development	3	4.84E-02
GOTERM_MF_FAT	GO:0030528~transcription regulator activity	83	1.88E-06
GOTERM_MF_FAT	GO:0016563~transcription activator activity	33	4.43E-06
GOTERM_MF_FAT	GO:0003700~transcription factor activity	52	5.01E-04
GOTERM_MF_FAT	GO:0019899~enzyme binding	32	9.62E-04
GOTERM_MF_FAT	GO:0043565~sequence-specific DNA binding	35	1.48E-03
GOTERM_MF_FAT	GO:0008134~transcription factor binding	30	2.80E-03
GOTERM_MF_FAT	GO:0003713~transcription coactivator activity	16	4.41E-03
GOTERM_MF_FAT	GO:0043236~laminin binding	4	4.66E-03
GOTERM_MF_FAT	GO:0003779~actin binding	20	1.05E-02
GOTERM_MF_FAT	GO:0050840~extracellular matrix binding	5	1.08E-02
GOTERM_MF_FAT	GO:0003677~DNA binding	95	1.35E-02
GOTERM_MF_FAT	GO:0003702~RNA polymerase II transcription factor activity	16	1.40E-02

GOTERM_MF_FAT	GO:0003712~transcription cofactor activity	21	1.57E-02
GOTERM_MF_FAT	GO:0046983~protein dimerization activity	28	1.88E-02
GOTERM_MF_FAT	GO:0051427~hormone receptor binding	8	2.46E-02
GOTERM_MF_FAT	GO:0043121~neurotrophin binding	3	2.60E-02
GOTERM_MF_FAT	GO:0008047~enzyme activator activity	19	2.61E-02
GOTERM_MF_FAT	GO:0060589~nucleoside-triphosphatase regulator activity	22	2.96E-02
GOTERM_MF_FAT	GO:0016564~transcription repressor activity	18	3.01E-02
GOTERM_MF_FAT	GO:0005083~small GTPase regulator activity	16	3.51E-02
GOTERM_MF_FAT	GO:0003705~RNA polymerase II transcription factor activity, enhancer binding	5	3.72E-02
GOTERM_MF_FAT	GO:0010843~promoter binding	6	3.76E-02
GOTERM_MF_FAT	GO:0035257~nuclear hormone receptor binding	7	3.94E-02
GOTERM_MF_FAT	GO:0005095~GTPase inhibitor activity	3	4.01E-02
GOTERM_MF_FAT	GO:0030695~GTPase regulator activity	21	4.21E-02
GOTERM_CC_FAT	GO:0044459~plasma membrane part	102	1.83E-05
GOTERM_CC_FAT	GO:0009986~cell surface	25	2.26E-04
GOTERM_CC_FAT	GO:0045202~synapse	24	7.22E-04
GOTERM_CC_FAT	GO:0009898~internal side of plasma membrane	21	2.10E-03
GOTERM_CC_FAT	GO:0009897~external side of plasma membrane	14	2.50E-03
GOTERM_CC_FAT	GO:0000267~cell fraction	51	2.88E-03
GOTERM_CC_FAT	GO:0005886~plasma membrane	142	5.31E-03
GOTERM_CC_FAT	GO:0005794~Golgi apparatus	41	8.40E-03
GOTERM_CC_FAT	GO:0005605~basal lamina	4	1.46E-02
GOTERM_CC_FAT	GO:0019717~synaptosome	8	1.64E-02
GOTERM_CC_FAT	GO:0019897~extrinsic to plasma membrane	6	1.90E-02
GOTERM_CC_FAT	GO:0030054~cell junction	26	1.95E-02
GOTERM_CC_FAT	GO:0005626~insoluble fraction	38	1.98E-02
GOTERM_CC_FAT	GO:0043005~neuron projection	19	2.09E-02
GOTERM_CC_FAT	GO:0034703~cation channel complex	10	2.19E-02
GOTERM_CC_FAT	GO:0030136~clathrin-coated vesicle	10	2.19E-02
GOTERM_CC_FAT	GO:0030055~cell-substrate junction	9	2.33E-02
GOTERM_CC_FAT	GO:0030665~clathrin coated vesicle membrane	6	2.39E-02
GOTERM_CC_FAT	GO:0016323~basolateral plasma membrane	13	2.49E-02
GOTERM_CC_FAT	GO:0030135~coated vesicle	11	2.66E-02
GOTERM_CC_FAT	GO:0005624~membrane fraction	36	2.91E-02
GOTERM_CC_FAT	GO:0005783~endoplasmic reticulum	41	3.44E-02
GOTERM_CC_FAT	GO:0000792~heterochromatin	5	3.76E-02
GOTERM_CC_FAT	GO:0031224~intrinsic to membrane	189	3.91E-02
GOTERM_CC_FAT	GO:0005925~focal adhesion	8	3.94E-02
GOTERM_CC_FAT	GO:0031012~extracellular matrix	18	4.16E-02
GOTERM_CC_FAT	GO:0015629~actin cytoskeleton	15	4.18E-02
GOTERM_CC_FAT	GO:0042995~cell projection	31	4.37E-02

GOTERM_CC_FAT	GO:0000307~cyclin-dependent protein kinase holoenzyme complex	3	4.40E-02
GOTERM_CC_FAT	GO:0005905~coated pit	5	4.69E-02
GOTERM_CC_FAT	GO:0005924~cell-substrate adherens junction	8	4.69E-02

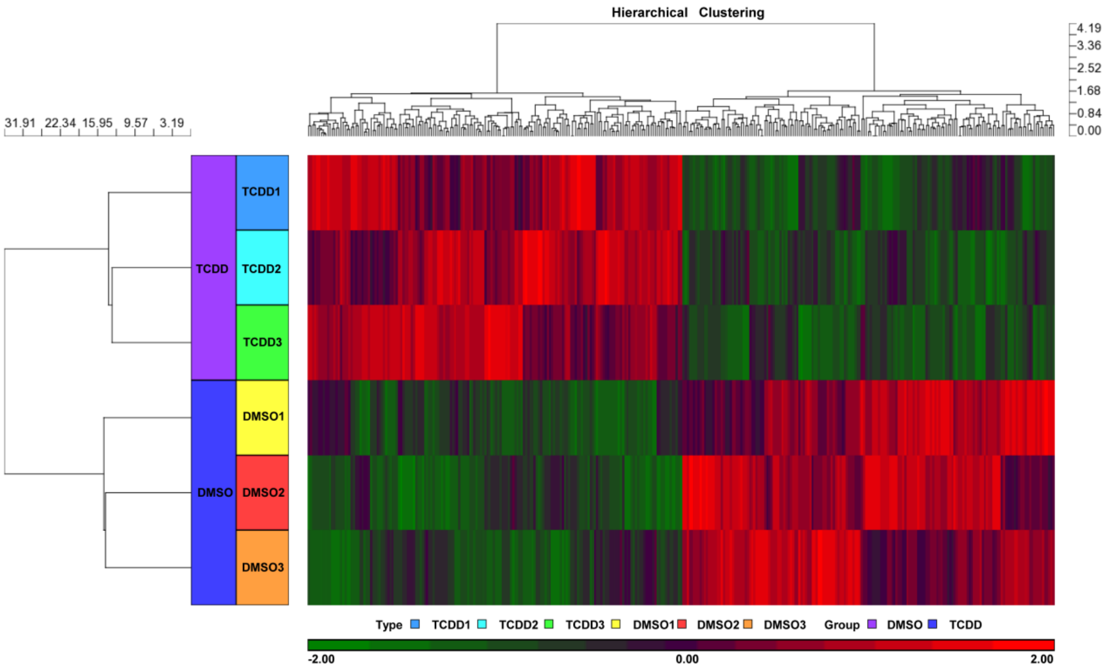

Supplementary figure 1. Differentially expressed miRs in response to dioxin in SK-N-SH cells. Cells were exposed to 10^{-10} M TCDD or 0.1% DMSO for 24 hr. The clustered heat map shows discrimination of all three replicates of TCDD (at 10^{-10} M) and DMSO treated groups. A dendrogram of the cluster analysis of miRs is shown.

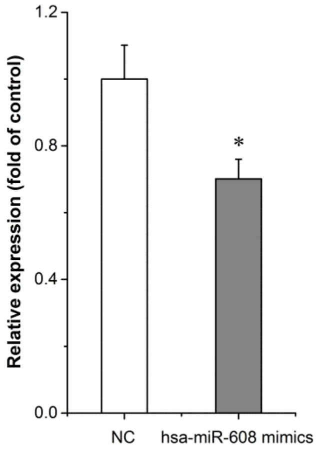

Supplementary figure 2. Hsa-miR-608 suppresses the expression of CDC42. SK-N-SH cells were transfected with hsa-miR-608 mimics or negative control (NC) for 24 hr. Total RNA was extracted for determination of the expression level of CDC42 mRNA. Quantitative PCR analyses were performed as mentioned in M & M. 18s ribosomal RNA was used as an internal control. Statistical analysis was done by t-test, and *$p < 0.05$ compared with negative control (NC) group. All values were expressed as mean ± S.E. (n=3) and each independent sample was detected in triplicate wells.
